# Nurse-Led Digital Intervention for Sodium Restriction in Chronic Kidney Disease: Mixed Methods Implementation Study

**DOI:** 10.2196/94330

**Published:** 2026-07-20

**Authors:** Yu Shi, Hongmei Peng, Youying Zhang, Jingyu Zhang, Yang Li, Shi Pu, Xiangchun Tang, Jinghong Zhao

**Affiliations:** 1 Department of Nephrology, Chongqing Key Laboratory of Prevention and Treatment of Kidney Disease, Chongqing Clinical Research Center of Kidney and Urology Diseases, The Second Affiliated Hospital (Xinqiao Hospital), Army Medical University (Third Military Medical University), 83 Xinqiao Zhengjie, Shapingba District, Chongqing, 400037, China, 86 13668007369; 2 State Key Laboratory of Ultrasound in Medicine and Engineering, Chongqing Medical University, Chongqing, China

**Keywords:** chronic kidney disease, sodium restriction, digital health, mixed-methods research, nurse-led digital intervention

## Abstract

**Background:**

Digital interventions can support sodium restriction in chronic kidney disease; yet, implementation and engagement barriers remain poorly understood.

**Objective:**

This mixed methods study evaluated a nurse-led digital program for sodium restriction, focusing on 3 key aspects: implementation, engagement, and patient experience.

**Methods:**

This study was initially designed as a randomized controlled trial but was revised to a mixed methods implementation study due to high rates of missing primary outcome data. Quantitative measures included 24-hour urinary sodium excretion (the primary outcome of the original randomized controlled trial), knowledge tests, blood pressure measurements, and quality-of-life assessments. Concurrently, we conducted in-depth qualitative interviews with a purposive sample of participants. Quantitative and qualitative data were collected in parallel, analyzed separately, and then integrated to provide a comprehensive understanding of participants’ experiences with the intervention.

**Results:**

Among 99 enrolled patients (46 in the enhanced digital intervention group and 53 in the minimal digital control group), the program led to a significant improvement in sodium-related knowledge as a secondary exploratory outcome (*P*=.005). However, the 24-hour urine collection completion rate was only 24.2% (n=24), precluding reliable conclusions regarding the primary outcome. Qualitative findings revealed a “measurement paradox,” in which the gold-standard assessment itself posed a major barrier to adherence. Integrated data illustrated a patient journey marked by early enthusiasm, followed by declining engagement due to digital fatigue, family resistance, and poor fit with daily life. This misalignment created a disconnect between acquired knowledge and measurable behavior change.

**Conclusions:**

A nurse-led digital intervention was associated with modest improvements in sodium knowledge in chronic kidney disease. However, success requires reducing the monitoring burden and improving real-world fit. The “measurement paradox” highlights the need for patient-centered outcome measures. We propose a “stage-matched intervention” framework to guide adaptive digital tools. Future research should prioritize multicenter pragmatic trials to bridge the knowledge-action gap.

## Introduction

Chronic kidney disease (CKD) affects millions worldwide, and high sodium intake accelerates disease progression [1-3]. Clinical guidelines recommend a daily sodium intake below 2.3 grams or 100 mmol to control blood pressure and preserve kidney function [4-6], but real-world adherence remains poor. Fewer than 30% of patients with CKD meet this target [7-9], revealing a critical knowledge-practice gap in disease management. As frontline providers, nurses are well positioned to leverage digital tools to improve sodium management [10-12]. To systematically address these self-care deficits, this study was guided by Orem’s Self-Care Deficit Nursing Theory, which posits that individuals require nursing support when their self-care capabilities are insufficient [13]. For patients with CKD, inadequate knowledge, poor adherence, and low self-monitoring skills represent key self-care deficits in sodium management. The nurse-led digital intervention was therefore designed to identify these deficits, provide compensatory support, and enhance self-care agency, fully aligning with the core logic of Orem’s theory. Accordingly, we developed a theory-driven, nurse-led mobile digital sodium restriction program embedded within our independently designed Su Yi app (Thinmed Medical Technology (Chongqing) Co, Ltd), integrating nursing expertise into patient education to address these self-care deficits [14,15].

While digital therapeutics have demonstrated benefits for chronic conditions such as hypertension [16-19], their application in CKD management remains nascent. Most existing studies rely on idealized randomized controlled trial (RCT) designs (Checklist 1) [7,20] that prioritize internal validity but overlook real-world implementation barriers, including digital fatigue, social resistance, and procedural burden. A prime example is the 24-hour urinary sodium test, the gold standard for estimating sodium intake, which imposes a considerable burden on patients due to its cumbersome nature [21]. Yet, its role as a primary end point is rarely reconsidered, creating a critical measurement paradox. Low adherence to such measures renders study outcomes inconclusive, hindering the identification of why interventions fail. Although some studies indicate that digital interventions can improve dietary knowledge among patients with CKD [22,23], the reasons why knowledge gains often do not translate into improved behavior or physiological outcomes remain unclear. Critically, large-scale failures in objective data collection often overshadow patients’ actual experiences with digital tools, obscuring systemic mismatches between intervention design and the realities of daily life.

Existing literature identifies a key paradox in digital health interventions. Patients often show significant improvements in health-related knowledge; yet, objective behavioral metrics, such as physiological assessment completion rates, remain consistently low. In sodium management programs specifically, knowledge acquisition does not reliably translate into adherence to urine collection protocols used to verify dietary changes. This discrepancy raises a critical question: why do theoretically effective interventions struggle to sustain in real-world settings? Possible explanations include intervention design flaws, insufficient patient motivation, or a fundamental misalignment between digital tools and patients’ daily experiences. The field currently lacks an in-depth understanding of the full engagement process among patients with CKD in digital sodium management. Without insights into patients’ emotional shifts, practical challenges, and evolving needs during intervention, there is a risk of repeatedly developing technically advanced but clinically impractical solutions. To address this gap, advanced practice nurses and clinical nurse researchers conducted a parallel mixed methods study integrating quantitative and qualitative approaches. Led by the nursing team, the intervention was designed and implemented to systematically evaluate both the effectiveness and patient experience of a nurse-developed digital sodium restriction program for patients with CKD.

Initially registered as an RCT (ChiCTR2200060627) with 24-hour urinary sodium as the primary outcome, this study was reconceptualized as an exploratory mixed methods implementation study due to 75.7% (75/99) missing data on the primary outcome. The objectives shifted from efficacy verification to exploring implementation barriers and patient engagement experiences. Guided by Orem’s Self-Care Deficit Nursing Theory, we hypothesized 2 core points. First, the nurse-led digital intervention may improve sodium-related knowledge but is likely to face high attrition in burdensome 24-hour urinary sodium collection due to mismatched self-care capabilities. Second, mapping longitudinal engagement trajectories is expected to identify the core implementation barriers, informing human-centered digital tool development and clarifying the knowledge-behavior disconnect. This study addresses a critical gap in digital nursing research by moving beyond traditional efficacy-focused RCTs to prioritize real-world implementation and user experience. Our findings offer practical guidance for designing adaptive, feasible digital interventions for CKD management.

## Methods

### Study Design and Setting

This study was prospectively registered as a parallel-group RCT in the Chinese Clinical Trial Registry (ChiCTR2200060627; registered on June 6, 2022). The initial design aimed to evaluate intervention efficacy, with 24-hour urinary sodium excretion as the primary outcome and secondary outcomes including sodium-related knowledge, blood pressure, and quality of life (QOL). A 3-month follow-up duration was selected based on previous digital health implementation studies in CKD and hypertension, which have demonstrated that 8 to 12 weeks is sufficient to detect changes in intermediate physiological and behavioral outcomes in real-world settings [7,17]. This time frame balances the need to observe meaningful behavior change while minimizing attrition in longitudinal implementation research.

During the 3-month interim review, only 24.2% (24/99) of participants completed valid 24-hour urine collections, resulting in 75.7% (75/99) missing data for the primary outcome, far below the prespecified acceptable threshold of 60%. Because increasing the sample size could not improve data completeness, the study design was formally revised from an RCT to a single-center, exploratory mixed methods implementation study [24-26].

This design adaptation was consistent with reporting guidelines for pragmatic implementation research. The original sample of 99 participants remained adequate for mixed methods inquiry, which typically requires at least 80 quantitative participants and 15 to 30 qualitative participants [27]. Accordingly, the analytical focus shifted from confirmatory efficacy testing to exploratory implementation outcomes. The core objective was revised to evaluate implementation processes, participant engagement experiences, and barriers to a nurse-led digital sodium restriction intervention in patients with CKD.

During the 3-month interim progress review conducted in November 2022, the completion rate for the prespecified primary outcome measurement was merely 24.2% (24/99), far below the expected level. Concurrently, notable participant attrition was observed in both groups. Given the poor feasibility of continuing the original primary outcome evaluation, our research team comprehensively assessed the actual research progress and field conditions. After obtaining formal approval from the ethics committee, we decided to abandon the planned primary outcome efficacy analysis, redefine this project as an exploratory mixed methods implementation study, and shift our core research focus to exploring intervention implementation processes and real-life patient experiences.

### Ethical Considerations

The research design and study protocol were approved by the Ethics Committee of the Second Affiliated Hospital of Army Medical University (approval number 2022-Research-040-01). The revised study design was subsequently reviewed and approved by the same ethics committee (approval number 2022-Research-515-01). All participants provided written informed consent prior to enrollment. All study procedures were conducted in accordance with the Declaration of Helsinki.

### Participant Recruitment and Screening

Eligible participants were recruited and screened by trained clinical nurses from inpatients admitted to the nephrology department between June 2022 and August 2022. Inclusion criteria were as follows: age 18 to 65 years; diagnosis of CKD stages 1‐5 [28], including those with hypertension; an estimated glomerular filtration rate stable over the past 3 months; 24-hour urinary sodium excretion >130 mmol/day at baseline; Barthel Index score ≥60; Mini-Mental State Examination (MMSE) score ≥24; proficiency in using smartphones and mobile applications; and willingness to participate. Exclusion criteria included the following: recent acute kidney injury; receipt of renal replacement therapy; major cardiovascular event within 6 months; uncontrolled hypertension (systolic blood pressure >160 mm Hg or diastolic blood pressure >100 mm Hg); prior participation in similar digital health programs; or any condition likely to interfere with study compliance.

### Randomization and Blinding

Eligible participants were randomly assigned (1:1) to either the intervention or control group using computer-generated random sequences in SPSS (version 24.0; IBM Corp). Allocation was concealed via sequentially numbered, opaque, and sealed envelopes prepared by an independent statistician. Due to the nature of the intervention, participants and the intervention delivery nurse could not be blinded. However, to minimize ascertainment bias, outcome assessors, laboratory technicians, and data analysts remained blinded to group allocation throughout the study. The 46:53 group size discrepancy resulted from random fluctuation of the computer-generated sequence with no manual modification. Such a degree of imbalance falls within the expected range of chance variation in simple randomization and does not compromise the validity of random allocation.

### Interventions

#### Control Group (Minimal Digital Intervention)

Participants received minimal digital intervention [29], which included a single educational session on sodium restriction and access to the Su Yi app—a self-developed mobile platform providing static educational content (1 video and 1 text document) [30]. They were prompted daily via the app to measure and log their morning blood pressure. Both groups used the Su Yi app; however, the intervention content and intensity differed between the groups.

#### Intervention Group (Enhanced Digital Intervention)

**Figure 1.**
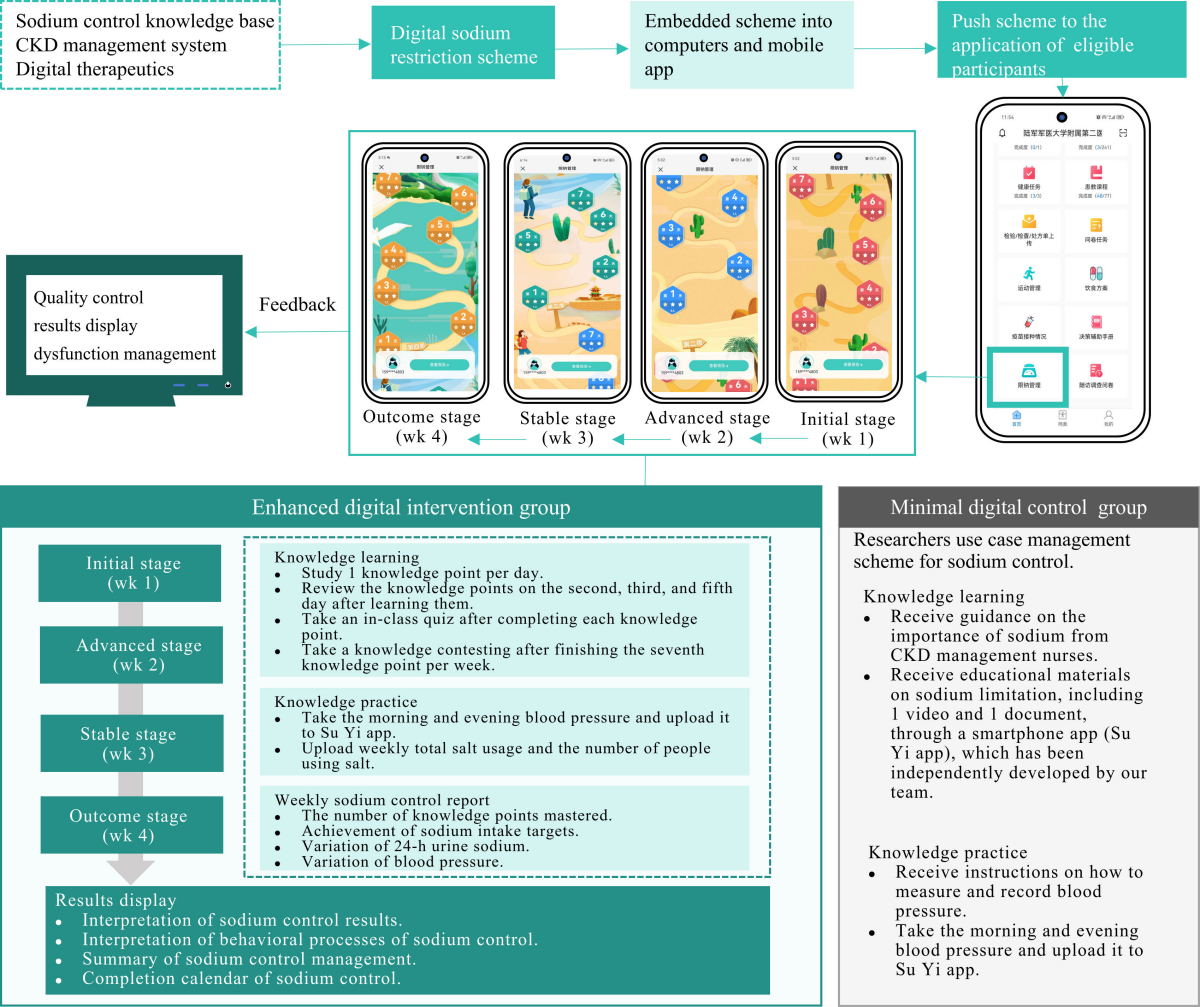
Formation and application process of the digital sodium control system. CKD: chronic kidney disease.

Participants received an enhanced digital intervention that was strictly grounded in Orem’s Self-Care Deficit Nursing Theory. The intervention followed three core theoretical components: (1) deficit identification through baseline knowledge assessment, (2) compensatory nursing support via structured education and gamified learning, and (3) self-care agency enhancement using personalized feedback and autonomous practice. The 4-week intervention was delivered in four progressive stages (Figure 1): (1) initiation (knowledge learning), (2) advancement (quizzes and peer competition), (3) stabilization (behavioral practice), and (4) outcome (personalized reporting). This staged structure directly operationalized Orem’s theoretical logic of identifying deficits, providing compensatory support, and promoting self-care capacity. Gamified modules, in-app quizzes, peer competition, and standardized behavioral tasks were integrated to enhance engagement. Upon completion of the 4-week intervention, each participant received a personalized report illustrating knowledge mastery, salt intake goal achievement, trends in 24-hour urinary sodium excretion, and blood pressure trajectories. All activities were completed autonomously via the app, minimizing clinical burden while maintaining consistent nursing oversight through remote monitoring.

Both groups attended a follow-up visit 3 months post intervention for outcome assessment.

### Sample Size Calculation

The sample size was calculated using PASS (version 15.0; IBM Corp) software based on the anticipated between-group difference in 24-hour urinary sodium excretion at the 3-month follow-up. Data from a previous randomized trial suggested a mean excretion of approximately 173.5 mmol/day in control groups and 147.5 mmol/day in intervention groups, indicating a potential difference of 26 mmol/day [31]. A common SD of 34.0 mmol/day was assumed based on the same study. Using a 2-sample, 2-tailed *t* test with α=.05% and 90% power, 37 participants per group were required. Accounting for an estimated 20% dropout rate, the final target sample size was set at 47 per group (total n=94).

### Data Collection Procedures

Quantitative data were collected at baseline (T0) and at the 3-month follow-up (T1). Outcomes of interest included 24-hour urinary sodium excretion [32] and blood pressure [33] as potential indicators of physiological response. Blood pressure was defined as the average of 3 consecutive office blood pressure measurements. Additional outcomes comprised sodium control knowledge, QOL, and laboratory parameters. Sodium control knowledge was assessed using a 10-item questionnaire developed by the research team. The questionnaire was based on CKD sodium restriction guidelines, refined through 3 rounds of expert review, with a content validity index of 0.92, and validated via pilot testing with 20 participants and a Cronbach α of 0.78, and the questionnaire is provided in Multimedia Appendix 1. QOL was measured using KDQOL-SF (Kidney Disease Quality of Life-Short Form) 1.3 [34], and laboratory parameters included serum creatinine, urea, uric acid, electrolytes, hemoglobin, and albumin. Adherence to 24-hour urine collection was anticipated to present challenges; therefore, this measure was analyzed exploratorily and interpreted cautiously, with due consideration of completion rates.

Qualitative data were collected through semistructured in-depth interviews with a purposive subsample of 23 participants. Participants who discontinued the intervention were not included due to practical constraints related to time and research resources. This subsample comprised only intervention completers; accordingly, all qualitative findings were limited to participants who completed the full intervention program.

All interviews were conducted in Mandarin Chinese by 2 nurse researchers with master’s degrees who had received systematic training in qualitative research (Checklist 2). These researchers were not involved in intervention delivery to minimize potential bias. Face-to-face interviews were conducted in the outpatient CKD management clinic, with each session lasting 20 to 40 minutes. All interviews were audio-recorded, transcribed verbatim, and anonymized. The transcripts were then translated into English using dual translation and back-translation to ensure linguistic and conceptual fidelity. Regular debriefing sessions were held throughout data collection to enhance reflexivity and analytic rigor. The interview guide was developed under the core framework of Orem’s Self-Care Deficit Nursing Theory, with the integration of key concepts from Empowerment Theory. The guide was designed to explore participants’ experiences across multiple dimensions, including task execution, emotional responses, barriers encountered, and evolving needs. Thematic analysis was used to analyze the interview transcripts. To ensure coding reliability, 2 researchers independently coded 20% of the data, yielding strong intercoder agreement with a Cohen κ value of 0.72. Discrepancies were resolved through consensus discussion. The finalized codebook, which included code definitions, thematic hierarchies, representative quotes, and decision rules, was then applied to the full dataset by the primary researcher. The codebook is provided in Multimedia Appendix 2. Sensitivity analyses were conducted to assess the potential influence of selection bias on qualitative interpretations. We hypothesized that participants who discontinued the intervention may hold distinct perspectives related to higher intervention burden, digital fatigue, and competing time demands. Such systematic differences may lead to overestimation of participant engagement and underestimation of real-world implementation barriers if noncompleters are excluded from analysis.

### Statistical and Analytical Approaches

#### Quantitative Analysis

During manuscript revision, we consulted a biostatistician regarding the handling of the substantial missing data in 24-hour urinary sodium excretion. It was determined that traditional inferential statistics (eg, *t* tests, ANOVA) would yield unreliable estimates due to severe attrition (75/99, 75.8% missing) and potential selection bias. Consequently, we have revised the manuscript to reflect a mixed methods evaluation framework, removed causal interpretations of urinary sodium outcomes, and focused the discussion on implementation insights and patient-reported experiences.

Continuous variables were tested for normality using the Shapiro-Wilk test. Normally distributed data were presented as mean (SD) and compared using independent samples *t* tests. Nonnormally distributed variables were reported as median (IQR) and analyzed using Mann-Whitney *U* tests. Categorical variables were expressed as frequencies (%) and compared using chi-square or Fisher exact tests. Intention-to-treat (ITT) analysis was applied to all primary outcomes. Statistical significance was set at *P*<.05. Analyses were performed using SAS software (version 9.4; SAS Institute Inc). Given the substantial missingness in 24-hour urinary sodium excretion (>60%), we carefully evaluated the data quality and implemented the following handling strategies: (1) ITT analysis was applied to all available data to maintain the randomized design’s integrity; (2) between-group inferential comparisons were not performed, even for completers, due to the high risk of selection bias; and (3) multiple imputation was not adopted as it would require untestable assumptions about the missing values, for example, missing at random, given the extreme missing rate. We interpret this outcome exploratorily, focusing on implementation insights rather than efficacy conclusions.

#### Qualitative Analysis

Transcripts were analyzed using thematic analysis guided by Empowerment Theory. A hybrid deductive-inductive approach was used: initial coding was informed by predefined domains (tasks, emotions, pain points, and needs), while emergent themes were iteratively refined. Two researchers independently coded 20% of the transcripts to assess reliability, achieving consensus through discussion. NVivo 12 (QSR International) was used for data management.

#### Integration of Mixed Methods

A convergent parallel mixed methods design was used in accordance with the GRAMMS (Good Reporting of A Mixed Methods Study) guidelines (Checklist 3) [35]. Quantitative and qualitative data were collected in parallel, analyzed independently, and subsequently integrated using a joint display matrix. Quantitative outcomes provided the primary analytical framework, while qualitative findings were used to explain, supplement, and contextualize the quantitative results. To ensure integration rigor, 2 researchers conducted independent interpretive integration; discrepancies were resolved through consensus discussions, and a third researcher reviewed the entire integration process.

## Results

### Participant Flow

**Figure 2.**
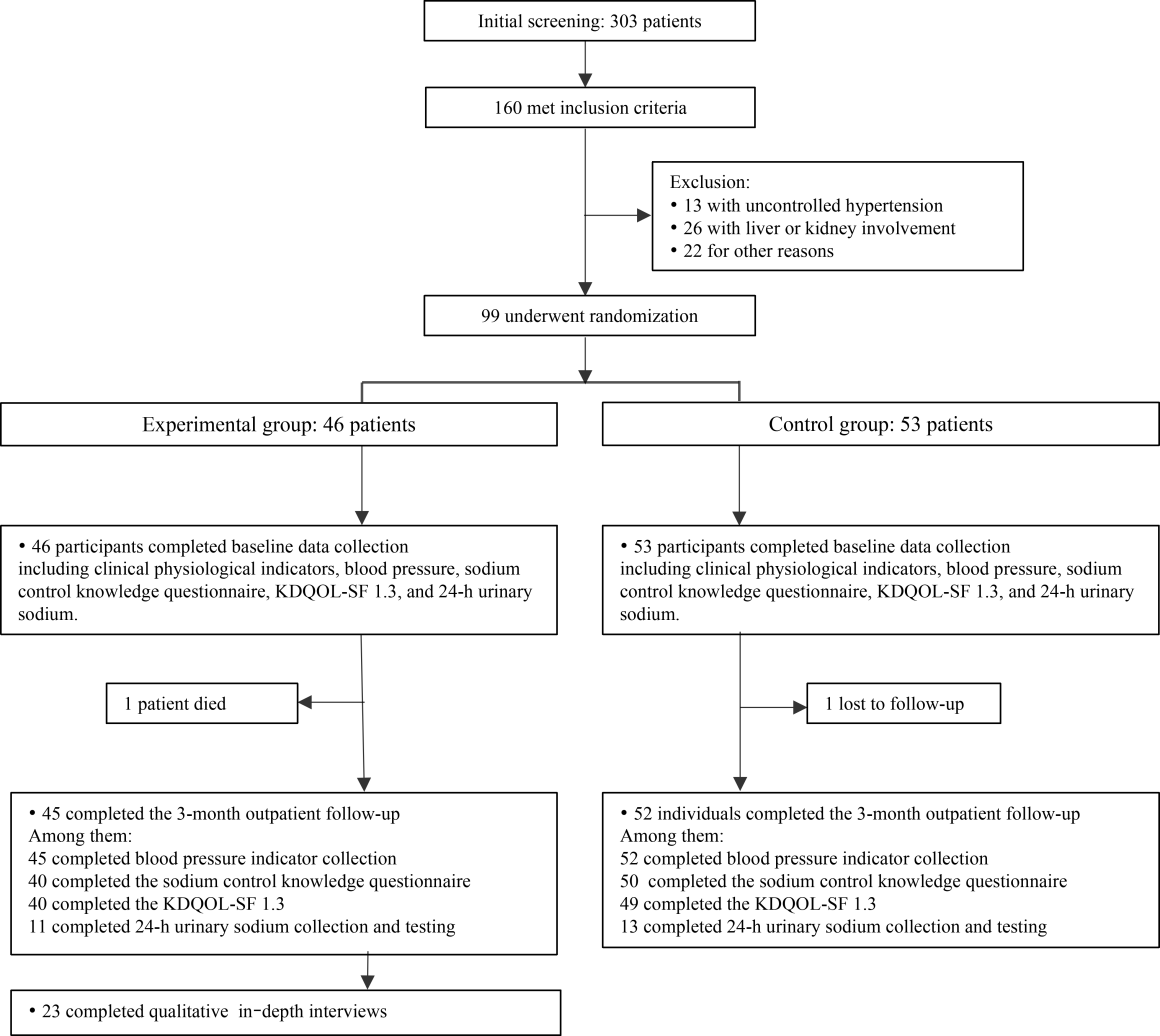
Flow diagram of participant enrollment. KDQOL-SF: Kidney Disease Quality of Life-Short Form.

Of 303 screened patients, 99 were randomized: 46 to the enhanced digital intervention group and 53 to the minimal digital control group. Baseline characteristics were similar between groups (Table 1), confirming balanced randomization. At the 3-month follow-up, 52 (98.1%) in the control group and 45 (97.8%) in the intervention group completed blood pressure and blood component analysis, with the exception of 1 death in the intervention group and 1 withdrawal in the control group for personal reasons. However, only 13 (24.5%) in the control group and 11 (23.9%) in the intervention group completed the 24-hour urine collection (Figure 2), resulting in an overall completion rate of 24.2% (24/99) for this outcome measure.

**Table 1.** Baseline demographic and clinical characteristics of participants (N=99).

Variables	Control (minimal digital; n=53)	Intervention (enhanced digital; n=46)	*z*	Chi-square (*df*)	*P* value
Age (y), median (IQR)	48 (37‐54)	44 (31-52)	−1.24	—^a^	.21
Sex, n (%)			—	1.56 (1)	.21
Male	32 (60.3)	22 (47.8)			
Female	21 (39.6)	24 (52.1)			
Educational level			—	1.47 (4)	.83
Primary education or below	11 (20.7)	7 (15.2)			
Junior middle school	19 (35.8)	15 (32.6)			
Senior high school	7 (13.2)	9 (19.5)			
Associate degree	8 (15.1)	6 (13.0)			
Bachelor’s degree or above	8 (15.1)	9 (19.5)			
Hypertension			—	1.51 (1)	.22
No	20 (37.7)	23 (50)			
Yes	33 (62.2)	23 (50)			
CKD^b^ stage			—	7.20 (4)	.13
1	13 (24.5)	20 (43.4)			
2	10 (18.8)	12 (26.1)			
3	13 (24.5)	6 (13.0)			
4	8 (15.1)	3 (6.5)			
5	9 (16.9)	5 (10.8)			
BMI (kg/m²), median (IQR)	24.6 (21.5-26.9)	24.0 (22.1-26.8)	−0.15	—	.88
Waist circumference (cm), median (IQR)	85 (78-94)	81 (77-92)	−1.55	—	.12
Urine protein to creatinine ratio (g/g), median (IQR)	1.8 (0.6-2.4)	1.2 (0.3-3.9)	−0.52	—	.60
Serum creatinine (μmol/L), median (IQR)	146.7 (85.3-315.5)	94.3 (94.3-56.6)	−1.90	—	.06
Urea (mmol/L), median (IQR)	9.2 (5.8-12.8)	6.1 (4.6-11.7)	−1.44	—	.15
Uric acid (μmol/L), median (IQR)	392.3 (329.8-482.6)	400.3 (308.0-481.0)	−0.69	—	.49
Glucose (mmol/L), median (IQR)	5.2 (4.7-5.6)	5.2 (4.8-5.9)	0.29	—	.78
Serum sodium (mmol/L), median (IQR)	139.0 (137.6-140.2)	138.6 (137.6-140.0)	−0.85	—	.40
Serum potassium (mmol/L), median (IQR)	4.1 (4.0-4.5)	4.1 (3.9-4.4)	−0.46	—	.64
Serum phosphate (mmol/L), median (IQR)	1.2 (1.0-1.3)	1.2 (1.0-1.3)	−0.29	—	.77
Serum calcium (mmol/L), median (IQR)	2.2 (2.1-2.3)	2.2 (2.1-2.3)	−1.21	—	.22
Total protein (g/L), median (IQR)	65.1 (59.1-72.4)	65.9 (58.1-73.6)	−0.14	—	.89
Hemoglobin (g/L), median (IQR)	126.0 (113.0-142.0)	129.0 (106.0-138.0)	−0.40	—	.80
Triglycerides (mmol/L), median (IQR)	1.8 (1.2-2.8)	1.6 (1.3-2.2)	−0.40	—	.69
Total cholesterol (mmol/L), median (IQR)	4.9 (4.4-6.0)	5.0 (4.1-6.0)	＜0.01	—	>.99
Systolic blood pressure (mm Hg), median (IQR)	130 (116-148)	127 (115-145)	−0.07	—	.95
Diastolic blood pressure (mm Hg), median (IQR)	82 (77-93)	82 (75-90)	−0.24	—	.81
Heart rate (beats/min), median (IQR)	82 (80-87)	81 (78-85)	−0.84	—	.40
Urinary sodium (mmol/24 h), median (IQR)	156.3 (141.4-184.6)	166.3 (140.8-206.7)	−0.84	—	.42
Sodium control knowledge score	3 (2-5)	3 (2-5)	1.13	—	.26
SF-36^c^, median (IQR)	2295 (2025-2500)	2247 (2247-1935)	−0.34	—	.73
KDTA^d^, median (IQR)	3126 (2673-3440)	3165 (2758-3483)	0.29	—	.77

a —: not applicable.

b CKD: chronic kidney disease.

c SF-36: 36-Item Short Form Health Survey.

d KDTA: kidney disease targeted areas.

### Baseline Characteristics

Baseline demographic and clinical characteristics were balanced between groups (Table 1). The median age was 48 (IQR 37‐54) years in the control group and 44 (IQR 31-52) years in the intervention group (*P*=.21). There were no significant differences in sex distribution, CKD stage, comorbidities, or laboratory parameters, confirming successful randomization.

### Quantitative Outcomes

As shown in Table 2, the completion rate for 24-hour urinary sodium excretion was critically low (24/99, 24.2%), precluding reliable estimation of between-group differences or definitive conclusions about behavioral change. Among the small subset of completers (n=24), median sodium excretion appeared lower in the intervention group (97.3, IQR 73.7‐124.0) compared to controls (154.4 mmol/day, IQR 123.2‐190.9). However, inferential testing was not conducted due to insufficient power and selection bias concerns.

However, a significant improvement was observed in the secondary outcome of sodium control knowledge: intervention group participants scored 8 (IQR 7-8) versus 6 (IQR 6-8) in controls (*z*=2.84, *P*=.005). No significant differences were found in blood pressure, QOL (36-Item Short Form Health Survey [SF-36] and kidney disease targeted areas), or other physiological markers.

**Table 2.** Analysis of outcomes at 3-month follow-up by study group (N=97)^a^.

Outcomes	Control (minimal digital; n=52)		Intervention (enhanced digital; n=45)		*z*	*P* value
	n^b^	Median (IQR)	n^b^	Median (IQR)		
Sodium control knowledge score	50	6 (6-8)	40	8 (7-8)	2.84	.005
SF-36^c^	49	2515 (2355-2640)	40	2535 (2315-2780)	1.06	.30
KDTA^d^	49	3375 (3048-3755)	40	3535 (3082-3939)	0.78	.44
Systolic blood pressure (mm Hg)	52	126 (112-141)	45	125 (112-139)	−0.22	.83
Diastolic blood pressure (mm Hg)	52	78 (72-88)	45	80 (73-88)	0.45	.66
Heart rate (beats/min)	52	80 (78-85)	45	80 (78-86)	0.38	.70

a Completion rate for 24-hour urinary sodium excretion was 24.2% (24/99). Between-group comparisons were not performed due to low adherence and risk of selection bias.

b Number of participants with available data for each outcome.

c SF-36: 36-Item Short Form Health Survey.

d KDTA: kidney disease targeted areas.

### Qualitative Findings

#### Overview of the Patient Journey Map

**Figure 3.**
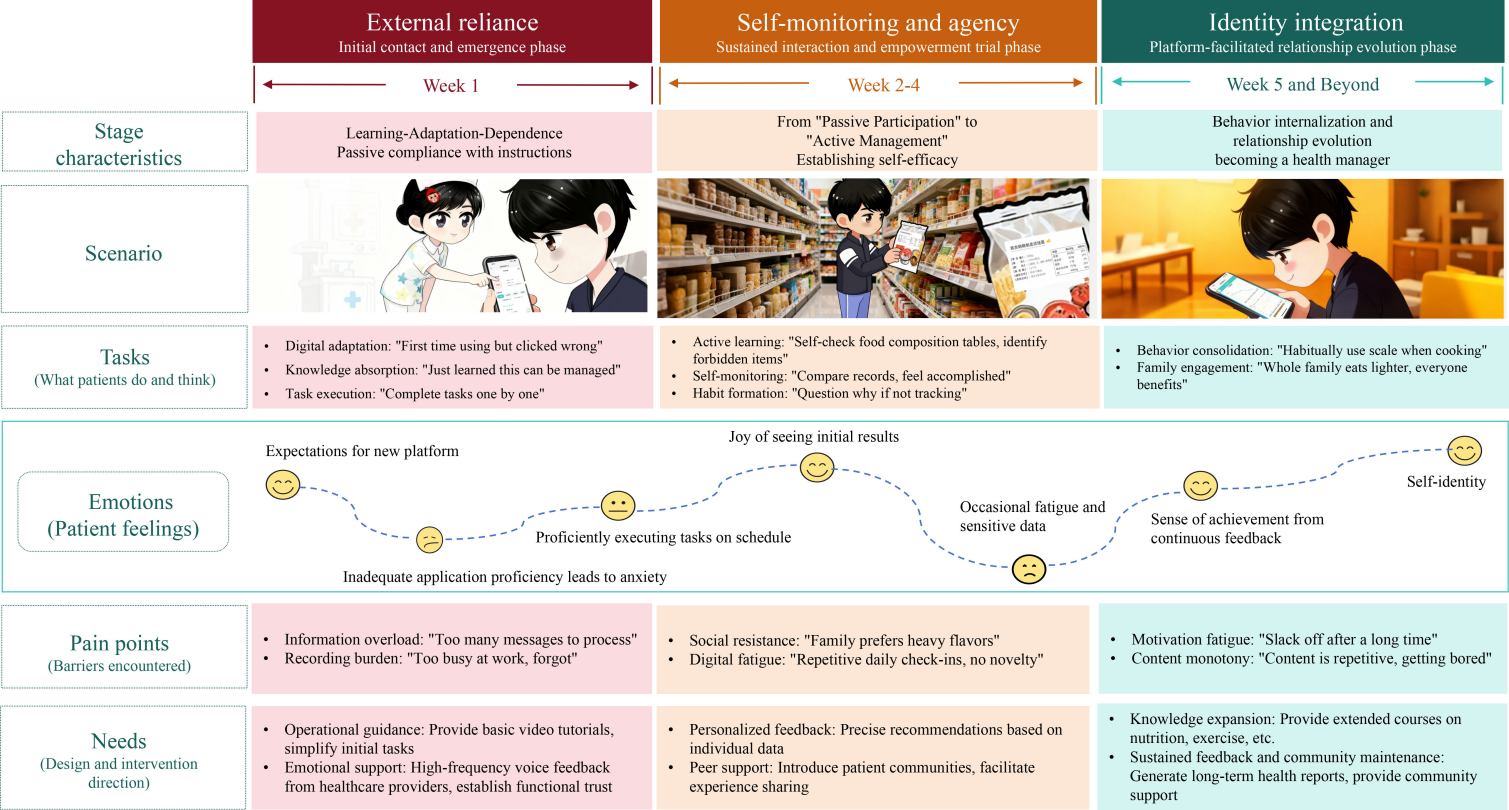
Patient empowerment journey among enhanced digital intervention completers.

Thematic analysis was conducted among 23 intervention completers; noncompleters were not interviewed due to resource constraints. Sensitivity analyses indicated that the exclusion of noncompleters may reduce the representativeness of the patient journey map. Consequently, all findings are restricted to participants who completed the enhanced digital intervention. Data saturation was assessed retrospectively after coding all 23 interview transcripts and was reached at the 18th interview, with no new themes identified in the subsequent 5 interviews. The analysis revealed a dynamic patient journey, summarized in Table 3. A patient journey map typically consists of a horizontal axis (timeline) and a vertical axis (task axis). Guided by Empowerment Theory, this study divides the typical journey of patients with CKD in a digital sodium restriction intervention along the horizontal axis into three phases: (1) initial contact and expectation phase (week 1); (2) sustained interaction and empowerment phase (weeks 2‐4); and (3) platform-facilitated relationship evolution phase (week 5 and beyond). The vertical axis encompasses 4 dimensions—tasks, emotions, pain points, and needs—yielding a total of 28 themes. These themes capture the realities, emotional shifts, barriers, and needs faced by patients at each stage of the digital sodium restriction intervention, comprehensively mapping the storyline across different dimensions throughout each phase. This reveals the dynamic evolution of patients from adherence to self-empowerment during the digital sodium restriction intervention, ultimately forming a typical patient journey map for patients with CKD in such interventions, as shown in Figure 3.

**Table 3.** Participant characteristics (qualitative study).

Number	Sex	Age (y)	Educational level	Profession	CKD^a^ stage
P1	Female	43	Primary education or below	Unemployed	5
P2	Male	52	Junior middle school	Farmer	2
P3	Female	29	Bachelor’s degree or above	Office worker	1
P4	Female	24	Associate degree	Professionals	1
P5	Female	37	Bachelor’s degree or above	Civil servant	1
P6	Male	61	Bachelor’s degree or above	Professionals	5
P7	Male	38	Junior middle school	Migrant worker	1
P8	Female	55	Senior high school	Retired	1
P9	Female	31	Bachelor’s degree or above	Civil servant	1
P10	Female	56	Primary education or below	Office worker	2
P11	Female	22	Junior middle school	Office worker	1
P12	Male	45	Associate degree	Civil servant	2
P13	Female	45	Junior middle school	Unemployed	3
P14	Female	28	Bachelor’s degree or above	Unemployed	1
P15	Male	47	Senior high school	Migrant worker	2
P16	Female	24	Junior middle school	Office worker	1
P17	Female	47	Junior middle school	Unemployed	1
P18	Male	43	Junior middle school	Migrant worker	3
P19	Male	27	Associate degree	Civil servant	4
P20	Male	50	Senior high school	Migrant worker	4
P21	Male	26	Associate degree	Professionals	1
P22	Female	37	Bachelor’s degree or above	Teacher	2
P23	Male	20	Associate degree	Student	1

a CKD: chronic kidney disease.

#### Initial Contact and Expectation Phase (Learning-Adapting-Depending)

Participants described initial excitement and hope (*“*I wanted to try anything that could help my kidneys*”* [P5/13]), which was quickly tempered by information overload and technical anxiety (*“*So many messages… I didn’t know what to do first*”* [P7]). Key barriers included difficulty navigating the app interface and uncertainty about correct data entry. Emotional dependency on nurse feedback emerged as a critical motivator for early adherence.

#### Sustained Interaction and Empowerment Phase (From Passive to Active Management)

As familiarity grew, participants transitioned from passive compliance to active self-monitoring and habit formation. Many reported checking food labels spontaneously (*“*Now I always look at the nutrition facts*”* [P5/7/21]) and using salt substitutes. Personalized feedback and peer comparison features enhanced motivation. However, digital fatigue (*“*It felt repetitive after a while*”* [P18]) and social resistance (*“*My family said the food was too bland*”* [P1/9]) emerged as significant challenges.

#### Platform-Facilitated Relationship Evolution Phase (Identity Integration)

In later stages, participants began identifying as health managers rather than passive patients. They shared knowledge with family members (*“*My kids now remind me not to eat pickles*”* [P13/16/20]) and advocated for healthier meals. Despite these positive shifts, motivational decay and content monotony threatened long-term engagement. Participants expressed strong desires for expanded educational content (eg, protein and potassium management) and ongoing community support.

### Mixed Methods Integration

Integrated analyses were performed using a joint display matrix (Table 4) following GRAMMS guidelines for mixed methods reporting. Three patterns of integration were identified. First, convergent findings demonstrated significant improvements in sodium-related knowledge in the enhanced digital intervention group (*z*=2.84, *P*=.005). These quantitative improvements were supported by qualitative accounts of clear, structured learning and enhanced confidence in sodium management, providing consistent support for the improvement in knowledge gains observed in the quantitative analysis. Second, supplementary findings highlighted a critical measurement paradox: completion of 24-hour urinary sodium collection was only 24.2% (24/99). Qualitative data further identified substantial procedural burden and poor compatibility with daily routines as key barriers, indicating that the gold-standard assessment itself impeded adherence. Third, explanatory findings clarified the absence of significant between-group differences in blood pressure or QOL (*P*>.05). Identified barriers, including family resistance, digital fatigue, and limited long-term support, explained why improvements in knowledge did not translate into measurable changes in clinical or patient-reported outcomes. No divergent findings were observed. The integrated synthesis indicates that although the intervention improved sodium-related knowledge, translation into behavioral and clinical changes was limited by implementation barriers and measurement burden.

**Table 4.** Joint display matrix for mixed methods integration.

Quantitative outcome	Qualitative finding	Integration type	Integrated interpretation
Sodium control knowledge: intervention 8 (7-8), control 6 (6-8); *z*=2.84, *P*=.005	Participants reported clear learning, easy-to-understand modules, and increased confidence in sodium management.	Convergence	The intervention effectively improved sodium-related knowledge, consistent with participant experience.
24 h urine completion: 24.2% (24/99); 75.7% (75/99) missing	Participants reported high burden, time intensity, and poor fit with daily life; urine collection was the top barrier.	Supplementation	Low completion is explained by substantial patient burden, revealing a “measurement paradox” in gold-standard outcome assessment.
BP^a^ or QoL^b^: no significant between-group differences (*P*>.05)	Barriers included family resistance, digital fatigue, repetitive tasks, and insufficient long-term support.	Explanation	Neutral clinical outcomes are explained by real-world implementation barriers that prevented knowledge from translating to behavior change.

a BP: blood pressure.

b QOL: quality of life.

## Discussion

### Principal Findings

This study found that the nurse-led digital intervention improved participants’ sodium-related knowledge as a secondary exploratory outcome. However, the completion rate of the primary outcome (24-h urinary sodium collection) was only 24.2% (24/99), which precluded evaluation of the intervention’s true efficacy. These findings are consistent with Orem Self-Care Deficit Nursing Theory. Qualitative interviews identified a 3-phase patient engagement journey: initial enthusiasm, active participation, and subsequent disengagement driven by digital fatigue and family barriers. Overall, while digital interventions can enhance health knowledge, high-burden assessment tools and poor adaptation to daily life significantly hinder sustained behavioral changes.

### From Information Delivery to Multidimensional Empowerment, Bridging the Knowledge-Action Gap

The improvement in knowledge without obvious behavioral changes supports Orem theory, which holds that self-care agency requires adequate knowledge, skills, motivation, and environmental support [36-39]. This reflects a typical knowledge-action gap. Digital tools can efficiently disseminate standardized health information on a large scale, but knowledge alone cannot drive long-term behavioral changes [40-43]. Sodium restriction is affected by family dynamics, cooking habits, and taste preferences—factors far more complex than simple information delivery [44,45]. Therefore, future interventions should shift from one-way knowledge popularization to multidimensional empowerment, comprehensively addressing both structural and interpersonal barriers [46-48].

### Selecting Scientifically Sound and Feasible Outcome Measures

This study revealed a measurement paradox. Although 24-hour urine collection is the gold standard for sodium intake assessment [49], its complicated operation and heavy burden lead to low patient adherence and high measurement error [50,51]. This phenomenon aligns with Orem's view that self-care interventions must match patients’ actual capabilities, which limits its application in clinical practice and research. By contrast, the spot urine sodium-to-creatinine ratio is simple to operate and has been validated to predict disease progression in multiple studies [52,53]. AI-based real-time urine testing may balance accuracy and convenience; however, its reliability across different patient groups requires further verification before large-scale application [54-56]. We therefore suggest researchers prioritize outcome measures that offer both scientific rigor and real-world feasibility, rather than pursuing methodological purity at the expense of practicality, to improve the clinical translation of research findings.

### Constructing a Dynamic, Stage-Matched Intervention Framework

Family resistance, lifestyle constraints, and digital fatigue are key barriers to long-term intervention effects [57-59]. Digital health success relies on continuous patient engagement rather than short-term adoption [60-62]. Based on Orem's self-care theory, we propose a stage-matched intervention framework that includes 3 core components: deficit identification, compensatory support, and self-care capacity improvement [63]. In the initial stage, medical staff identify patients’ knowledge gaps and practical obstacles to build trust. In the middle stage, personalized digital guidance is used to improve patients’ self-efficacy and bridge capability gaps. The later stage helps patients maintain healthy behaviors through community interaction and independent self-care management. This framework transforms static, one-size-fits-all intervention modes into adaptive, dynamic support that addresses the multidimensional self-care deficits in CKD sodium management. Future mixed methods studies should further explore the mechanisms of patient engagement to inform the development of truly patient-centered digital intervention solutions.

### Strengths and Limitations

This study adopted a parallel mixed methods design. Combining quantitative data and qualitative narratives clarifies intervention effects and their underlying mechanisms—an integrated approach that remains underused in digital therapeutics research. Conducted in routine outpatient settings without additional resource input, the study demonstrates good ecological validity and practicality. The proposed patient empowerment pathway also provides a new perspective for exploring digital health engagement among patients with CKD.

Several limitations of this study should be acknowledged. First, the 24.2% (24/99) completion rate of 24-hour urine collection resulted in massive missing data, rendering ITT analysis and primary outcome testing invalid. Consequently, the true intervention effect could not be confirmed. Second, qualitative participants were limited to intervention completers, which may introduce selection bias, potentially overestimating engagement and underestimating implementation barriers, even after sensitivity analysis. Third, the single-center setting and small sample size limit the generalizability of findings across geographic, demographic, and statistical dimensions. Fourth, we did not analyze outcome differences across CKD stages 1 to 5, further limiting the applicability of the results.

### Conclusions

This exploratory mixed methods study indicated that the nurse-led digital sodium restriction intervention was associated with modest gains in participants’ sodium-related knowledge as a secondary outcome. However, this study encountered prominent practical difficulties in implementing high-load outcome monitoring, with the completion rate of 24-hour urine collection reaching only 24.2% (24/99). The misalignment between idealized trial protocols and patients’ daily realities underscores the need for more feasible, patient-centered outcome measures and adaptive intervention designs. Rather than drawing conclusions about efficacy, this study provides critical insights into implementation barriers and informs the development of future person-centered digital tools. Digital health interventions may be improved via technical enhancements and better adaptation to patients’ daily lives. Future research should use implementation science and mixed methods to explore intervention mechanisms, thereby narrowing the gap between health knowledge and self-management in chronic disease management.

## Supplementary material

10.2196/94330 Multimedia Appendix 1 Sodium salt knowledge questionnaire.

10.2196/94330 Multimedia Appendix 2 Patient interview guide and coding codebook.

10.2196/94330 Checklist 1 CONSORT-eHEALTH checklist (V 1.6).

10.2196/94330 Checklist 2 COREQ checklist.

10.2196/94330 Checklist 3 GRAMMS checklist.

## Abbreviations

CKD: chronic kidney disease
GRAMMS: Good Reporting of A Mixed Methods Study
ITT: intention-to-treat
KDQOL-SF: Kidney Disease Quality of Life-Short Form
MMSE: Mini-Mental State Examination
QOL: quality of life
RCT: randomized controlled trial
SF-36: 36-Item Short Form Survey
