# Supplementary material for: Nurse-Led Digital Intervention for Sodium Restriction in Chronic Kidney Disease: Mixed Methods Implementation Study
Source: JMIR Nurs. 2026 Jul 20;9:e94330. doi: 10.2196/94330 (PMC13384475; doi:10.2196/94330)
Supplement: Multimedia Appendix 1 [file nursing-v9-e94330-s001.pdf]

Supplementary Material 1. Sodium Salt Knowledge Questionnaire

Instructions: This questionnaire is designed to assess your current knowledge of sodium salt intake. Please mark the option that best reflects your understanding with a "√".

1. Do you know how many grams the most common table salt package weighs on the market?
   A. 200g
   B. 400g
   C. 500g
   D. Not sure
2. Do you know how much sodium is in 1 gram of table salt?
   A. 0.2g
   B. 0.3g
   C. 0.4g
   D. Not sure
3. In daily life, what type of salt is most commonly consumed ?
   A. Iodized salt
   B. Zinc-enriched salt
   C. Selenium-enriched salt
   D. Bamboo salt
4. What is the main chemical component of salt?
   A. Potassium chloride
   B. Potassium iodide
   C. Sodium chloride
   D. Iodine
5. The World Health Organization (WHO) recommends that each person's daily salt intake should be:
   A. < 2g/day
   B. < 3g/day
   C. < 5g/day
   D. < 6g/day
6. What is hidden salt?
   A. In daily life, salt is rarely used, but the portion of salt still unconsciously consumed from other foods.
   B. Hidden salt means not eating salt at all.
   C. Hidden salt means not eating raw salt, and it must be cooked.
   D. Hidden salt refers to soy sauce
7. Which of the following food categories contains hidden salt?
   A. Fruit
   B. Vegetables
   C. Dessert
   D. Meat

8. Excessive salt intake may lead to the following diseases:
    A. High blood pressure
    B. Lumbar disc protrusion
    C. Hyperthyreosis
    D. Bone fractures
9. Drinking more water helps your body get rid of excess sodium. How much water should a healthy adult drink each day?
    A. 1500-2000ml
    B. 1000ml
    C. 3000ml
    D. 500ml
10. 15ml of soy sauce is approximately equal to how many grams of salt?
    A. 3g
    B. 5g
    C. 10g
    D. 12g
