# Supplementary material for: Nurse-Led Digital Intervention for Sodium Restriction in Chronic Kidney Disease: Mixed Methods Implementation Study
Source: JMIR Nurs. 2026 Jul 20;9:e94330. doi: 10.2196/94330 (PMC13384475; doi:10.2196/94330)
Supplement: Multimedia Appendix 2 [file nursing-v9-e94330-s002.docx]

**Supplementary Material 2**

**2.1 Patient Interview Guide**

| Number | Age | Gender |
| --- | --- | --- |
|  |  |  |
| Marriage status | Highest qualification | Profession |
|  |  |  |
| **I. Warm-up and Basic Information Guidance** | | |
| 1. What was the reason for your participation in this digital sodium restriction intervention project? 2. Before the intervention, what expectations did you have for sodium-restricted diet for kidney disease and online intervention methods? | | |
| **II. Initial Contact and Expectation Phase (Week 1) Related Questions** | | |
| 1. What was your first feeling when you first came into contact with the intervention platform (tool)? Were there any difficulties in operation? 2. What specific problems did you encounter when implementing the sodium-restricted diet and using the platform in the first week? 3. What was your emotional state in the initial stage of the intervention?   4. In the initial stage, what was the core need you most hoped the medical staff or platform would solve? | | |
| **III. Sustained Interaction and Empowerment Phase (Weeks 2-4) Related Questions** | | |
| 1. After using the platform for 2-4 weeks, what obvious changes did you have in sodium-restricted diet and platform operation? 2. Did you start to take the initiative to adjust the low-sodium diet plan? What were the specific manifestations? 3. What was the biggest pain point you encountered in the sodium restriction intervention during this phase? 4. Did the platform services enhance your ability to manage your condition independently?   5. Compared with the initial stage, what changes have taken place in your mood and mentality? | | |
| **IV. Platform-Facilitated Relationship Evolution Phase (Beyond Week 4) Related Questions** | | |
| 1. After 4 weeks of intervention, what changes have taken place in your views on the platform and sodium restriction management? 2. During the long-term adherence process, what changes have taken place in your communication and dependence relationship with medical staff and the platform? 3. Can you adhere to a sodium-restricted diet independently for a long time? Are there any unmet needs?   4. In the later stage of the intervention, were there any new difficulties or concerns? | | |
| **V. Summary and Sublimation** | | |
| 1. During the intervention, what was the biggest growth in your ability to manage your condition? 2. What impact will this intervention have on your long-term management of kidney disease and adherence to a low-sodium diet in the future? | | |

Interviewer : Date:

**2.2 Thematic Analysis Coding Codebook**

For Nurse-Led Digital Sodium Restriction Intervention in CKD Patients

Date of Development: [2023.01.21]

Based on: Empowerment Theory + Semi-structured In-depth Interview Guide for the study

Applicable Scope: Transcripts of 23 CKD patients who completed the full digital sodium restriction intervention (purposive subsample)

Coding Hierarchy: 3-level coding (Core Theme → Sub-theme → Code), consistent with the 3-phase interview framework; coding strictly follows the principle of "data-driven inductive extraction + theory-guided deductive calibration"

**1. Coding Instructions**

1.1 Coding personnel: Two nurse researchers with master’s qualifications and trained in qualitative research methods; the primary researcher is responsible for applying the finalized codebook to the full dataset.

1.2 Coding rules: Adhere to the principle of semantic consistency; each coding label corresponds to the core semantic meaning of the patient’s expression, and the same semantic content is uniformly coded with the same label.

1.3 Discrepancy resolution: For inconsistent coding judgments, resolve through group discussion of the research team until a consensus is reached; the discussion results are recorded and used to revise and optimize the codebook.

1.4 Exclusion criteria: Non-substantive oral expressions (e.g., "um", "well", "it’s okay"), irrelevant daily communication content, and repeated redundant descriptions that do not add new information are excluded from coding.

1.5 Anonymization rule: All interview quotes are marked with "P+number" (e.g., P1, P2) to protect patient privacy, and no personal identifying information is included.

**2. Coding Hierarchy, Definitions and Examples**

Core Theme 1: Initial Contact and Adaptive Experience (Week 1)

Definition: Patients’ subjective feelings, operational experience, diet implementation problems, emotional state and core demands in the first week of contacting the digital intervention platform and implementing sodium-restricted diet.

| **Sub-theme** | **Code** | **Code Definition** | **Typical Interview Examples (Anonymized)** |
| --- | --- | --- | --- |
| Tasks | Digital adaptation | Patients generally lack prior experience with digital intervention platforms upon initial contact, resulting in limited understanding of the operational procedures and functions. | P1:*"The first time I used it, I was scared I might tap on something wrong. My daughter had to walk me through it step by step."*  P6: *"This app keeps lagging. I don’t know if it’s my phone or what."*  P10: *"When I put my phone in senior mode, the screen doesn’t show everything — I still have to ask my kid for help."* |
|  | Knowledge enlightenment | Patients gradually recognize the diverse sources of high-sodium foods and the health hazards of excessive sodium intake, thereby developing an initial understanding of the concept of sodium restriction. | P2: *"Before, I just knew not to eat pickles. But now I finally get that even ham sausa*ges have sodium in them."  P6: *"I only understood after watching the video that there's this thing called hidden salt — a lot of f*oods actually contain salt."  P17: *"I know I need to cut down on salt. Keeping salt under control is good for your health."* |
|  | Task execution | Patients perceive daily check-ins and blood pressure uploads as tasks of “adhering to medical advice,” with their behavioral motivation primarily driven by external instructions. | P1: *"The nurse said I need to study and answer questions every day. So I just do it — when it says answer questions, I answer questions, and I finish them one by one."*  P15:*"I don't really know what's the point of answering those questions, but I still complete the task every day. I'm afraid that if I don't do it, it might affect the doctor's judgment."* |
| Emotions | Expectancy | The digital management model brings a sense of novelty and hope, and patients exhibit positive expectations regarding improvement in their condition. | P5: *"The nurse said it can help me eat more scientifically and that it's good for my condition, so I thought I just had to give it a try."*  P12: *"I hope this app can help bring my blood pressure down so I don't need to increase my medication."* |
|  | Anxiety and unease | Patients worry about making errors in usage or receiving negative evaluations, manifesting technology-related anxiety and a sense of role uncertainty. | P11: *"You have to upload your blood pressure, and I'm worried that if I upload the wrong numbers, they'll think I'm not taking it seriously."*  P7: *"Sometimes after watching the video, I keep getting the answers wrong on the quiz. It's embarrassing—people might laugh at me."* |
|  | Establishment of a sense of belonging | Patients demonstrate a high level of trust in healthcare professionals, regarding their feedback as the primary source of psychological support. | P3: *"Every time the nurse praises me, I feel like I've done the right thing."*  P6: *"Even just a couple of words back from the doctor make me really happy."* |
| Pain points | Information overload | Patients are exposed to a large amount of health information in a short time. The fragmented content and frequent push notifications cause confusion and cognitive fatigue. | P7: *"There are too many messages — sometimes I can't keep up. I have to learn something new every day, and still go over what I learned the day before."*  P10: *"I don't know which content matters most. I'm afraid I'll miss something important."* |
|  | Recording difficulties | The recording process is complex and does not fit well with patients' daily routines, resulting in some patients experiencing performance pressure and frustration. | P4: *"You have to record your blood pressure every day. But sometimes when I go to work and don't have the blood pressure monitor with me, I just can't do it."*  P16: *"I only did it for a few days when I was in the hospital. After that, I stopped. I'm too busy with my business — I get caught up and just forget."*  P20: *"That urine test — the 24-hour urinary sodium — who can actually do that? We're working. How is that even possible?"* |
| Needs | Operational guidance | Patients express a general need for more intuitive and systematic operational training to reduce usability barriers and boost confidence in digital interventions. | P18: *"I feel like it's kinda complicated. The simpler, the better. If there was a video walking me through it step by step, that'd be great."*  P2: *"I didn't get much schooling. It'd be best if someone could just show me how to do it once — I pick things up easier that way."* |
|  | Emotional support | Patients seek attention and positive reinforcement from healthcare providers to relieve anxiety and sustain motivation for continued participation. | P10: *"The nurse checks in on me every day and gives me reminders — that really makes me want to keep at it."*  P7:*"Just getting a reply from the doctor makes me feel like I'm not forgotten. That's what keeps me going."* |

Core Theme 2: Sustained Interaction and Empowerment Experience (Weeks 2-4)

Definition: Patients’ changes in platform operation ability and sodium-restricted diet implementation after 2-4 weeks of intervention, active adjustment behavior of diet plan, pain points in intervention, self-management ability improvement and emotional and mental changes.

| **Sub-theme** | **Code** | **Code Definition** | **Typical Interview Examples (Anonymized)** |
| --- | --- | --- | --- |
| Tasks | Active learning | Patients gradually shift from passive acceptance to active exploration, developing a self-directed learning mode. | P8:*"These days I look up the sodium in foods myself and try to eat different things."*  P2: *"I even showed my partner how to cook with less salt — like, you can use a little vinegar to season things."* |
|  | Self-monitoring | Through data feedback from the platform, patients engage in self-correction, which enhances their behavioral awareness and sense of control. | P11: *"Every day I check my logs and see where I ate too much. Seeing that sodium curve drop — that feels really good."*  P4: *"I also pull up my blood pressure numbers and take a look — see if it's been high lately and how I'm doing with keeping it under control."* |
|  | Habit formation | Recording behaviors and sodium restriction gradually become integrated into daily life, leading to the establishment of stable health habits. | P7: *"Every morning as soon as I get up, I take my blood pressure. If I miss a day of checking in, I feel like something's off."*  P5:*"I bought one of those salt-control spoons online. Now when I cook, I just automatically watch how much salt I use."* |
| Emotions | Enhanced sense of competence | Continuous feedback fosters a sense of achievement, enabling patients to gradually build self-efficacy and confidence in their ability to manage their condition. | P11: *"I never thought I could actually watch what I eat — not like before when I was so careless. My last urine sodium was only around 30. When I left the hospital, it was over 100."*  P2: *"Anyway, I've learned a ton — like cutting back on salt, how much to put in cooking, all that. I've got it under control now. I'm pretty much off my meds, and all my numbers are better."* |
|  | Transient setbacks | Occasional deviations from the prescribed regimen occur in social or group dining contexts, leaving patients prone to feelings of guilt and self-blame. | P6: "Sometimes I have to go to dinners, eat out, and when I end up having too much salt, I just feel awful about it."  P10: "Once in a while I forget to log in, and then my record breaks. I feel like everything I did before was for nothing." |
|  | Self-motivation | Patients develop self-reward and competition mechanisms that reinforce sustained motivation for behavior change. | P16: *"I give myself little goals, and when I hit them, I reward myself."*  P4: "Seeing that I've logged in 20 days straight — that just makes me want to stick with it." |
| Pain points | Social resistance | Family dietary habits and social environments constrain the maintenance and implementation of sodium-restriction behaviors. | P9: *"My family prefers strong flavors — I'm the only one going light on salt. You can't cook just for me, you know."*  P5: *"When we eat out with others, everyone keeps telling me to eat more. I feel bad turning them down."* |
|  | Digital fatigue | Long-term repetitive logging and information reception lead to psychological burnout, causing patients to gradually lose their motivation and interest in active participation. | P18:*"It's just the same routine day after day. You've gotta log stuff every single day — sometimes multiple ti*mes — and it just gets old."  P11: *"There's so much stuff coming in, and you have to do lessons every day. When I see it all, I just lose the desire to even look."* |
| Needs | Personalized feedback | Patients expect to receive precise recommendations based on their individual data, so as to enhance the relevance and effectiveness of self-management. | P11: *"I wish it would show me exactly what I'm doing wrong, not just send these vague reminders."*  P2: *"If it could take my blood pressure trends into account and give me advice that's just for me — that would be great."* |
|  | Peer support | Patients look forward to exchanging experiences with fellow patients, seeking empathy and emotional encouragement. | P4: *"Seeing other people keep at it too — it just feels like we're all working on it together."*  P7: *"If everyone else is managing to log in every day, it makes me wanna do it too."* |

Core Theme 3: Platform-Facilitated Relationship Evolution and Long-Term Adherence Willingness (Beyond Week 4)

Definition: Patients’ cognitive changes to the digital platform and sodium restriction management after 4 weeks of intervention, changes in communication and dependence relationship with medical staff and the platform, long-term independent adherence ability, unmet needs and new difficulties/concerns in the later stage of intervention.

| **Sub-theme** | **Code** | **Code Definition** | **Typical Interview Examples (Anonymized)** |
| --- | --- | --- | --- |
| Tasks | Behavioral solidification | Sodium-restriction behaviors gradually become internalized as daily habits, with patients consciously adhering to healthy eating practices in their everyday lives. | P8: *"I think I already eat pretty light. I'm just used to cooking with less salt — I don't need reminding. Like the other day when we had fish out in the countryside — everyone said it wasn't salty, but to me it was way too salty and made me feel bad. So I guess my taste is just lighter than other people's now."*  P21: *"I used to never look at the nutrition labels when I bought stuff. But now I always check them."* |
|  | Family co-management | Patients integrate the principles of sodium restriction into household dietary management, leading to shared practices at the family level. | P1: *"With salt and all — my whole family eats light now. We try to keep things low-salt. Everyone in the family benefits from it.*"  P20: *"My child keeps telling me not to eat pickles and to follow the suggestions you gave me. That's what's helped me keep going."* |
| Emotions | Enhanced sense of control | Patients gain confidence in managing their disease through self-monitoring and experience a stable sense of self-efficacy. | P2: *"I used to depend on the doctor for everything. But now I've learned so much on my own — I can take care of myself."*  P7:*"This program is really about keeping sodium in check — since my blood pressure was a little high. Now that I've got it under control, my readings are pretty much normal."* |
|  | Self-identity | Patients begin to embrace the role of "health manager" as part of their self-concept, reflecting a process of role reconstruction. | P21: *"I've got salt knowledge down pat. I'm a 'sodium pro' now — people come to me for advice on what to eat."*  P1: *"Now I tell everyone I know — my friends, people around me — to cut back on salt. You don't need it as strong as before. Just a tiny bit of salty taste is fine."* |
| Pain points | Motivation decline | Prolonged intervention duration is associated with a progressive reduction in patients' adherence and self-monitoring engagement, reflecting characteristic behavioral fatigue. | P5: *"I was really into it at the beginning, but then it just became a pain."*  P3: *"If nobody's there to remind me, I'm more likely to forget. I guess over time you just get lazy about it."* |
|  | Content monotony | Infrequent content updates and substantial repetition within the platform dampen patients' learning drive, negatively affecting their continued involvement. | P21: *"There's not really much to learn — it's all the same stuff. After a while you just get sick of seeing it."*  P2: *"I've pretty much gone through everything on the app, and I still remember what I learned before. Without anything fresh, it's just not helpful anymore."* |
| Needs | Knowledge expansion | With the gradual internalization of sodium restriction, patients' health awareness broadens from isolated salt reduction to comprehensive disease management. | P18:*"Now on top of watching my salt, I also wanna know how much protein and potassium I should be eating."*  P9: *"For people with our condition, there's gotta be more to it than just cutting salt. I'd like to know about that stuff too."*  P22: *"I think it'd be even better if the app could cover exercise — like what kinds of exercises someone with nephrotic syndrome can do. That'd be awesome."* |
|  | Continuous feedback and community companionship | Patients expect the platform to provide ongoing data tracking and community-based support, in order to maintain their motivation for self-monitoring and sense of belonging. | P8: *"It's super important to me to see my progress over time — like my sodium dropping and my blood pressure staying steady. Those graphs just make everything feel worthwhile, and they really push me to keep at it."*  P9:*"Having other patients cheering each other on makes it harder to quit. When I see other people still hanging in there, it reminds me that I can't let myself slip either."* |

**3. Coding Decision Rules for Special Scenarios**

3.1 Cross-semantic content coding: If a patient’s single expression involves multiple coding labels (core themes/sub-themes/codes), all corresponding labels are marked, and the core semantic label is marked first (e.g., P1: "At first I was worried about tapping the wrong thing. Now I just do the daily questions — I know not to eat pickles." → simultaneously coded as "Digital adaptation", "Knowledge enlightenment", "Task executionl").

3.2 Vague semantic content coding: For patients’ vague and ambiguous expressions, combine the context of the whole interview to judge the core semantic meaning; if the context cannot be confirmed, mark it as "to be confirmed" and resolve it through research team discussion.

3.3 Repeated semantic content coding: For the same semantic content repeatedly expressed by the patient in different parts of the interview, only code it once, and do not repeat coding.

3.4 Negative expression coding: For patients’ negative expressions about the platform, intervention and diet implementation (e.g., dissatisfaction, complaint), code according to the core semantic meaning of the negative content, and do not set independent negative coding labels.
