# Supplementary material for: Nurse-Led Digital Intervention for Sodium Restriction in Chronic Kidney Disease: Mixed Methods Implementation Study
Source: JMIR Nurs. 2026 Jul 20;9:e94330. doi: 10.2196/94330 (PMC13384475; doi:10.2196/94330)
Supplement: Checklist 1 [file nursing-v9-e94330-s003.pdf]

CONSORT-EHEALTH v1.6 Checklist for Randomized Controlled Trials of Electronic Health Interventions

Manuscript Title: Nurse-Led Mobile Digital Intervention for Sodium Restriction in Chronic Kidney Disease: An Exploratory Mixed-Methods Study on Patient Journey and Implementation Barriers

Manuscript Number: 94330

Corresponding Author: Jinghong Zhao

Date Completed: 2026.04.08

Trial Registration Number: ChiCTR2200060627 (Chinese Clinical Trial Registry, registered on 06 June 2022)

Section/Topic	Item No.	CONSORT Checklist Item	EHEALTH Extensions (additions to, or clarification of the CONSORT item)	Study Response (Directly Aligned with Manuscript)
**TITLE & ABSTRACT**	1a	Identification as a randomized trial in the title	i) Identify the mode of delivery in the title. Preferably use “web-based” and/or “mobile” and/or “electronic game” in the title. Avoid ambiguous terms like “online”, “virtual”, “interactive”. Use “Internet-based” only if intervention includes non-web-based Internet components (e.g., email), use “computer-based” or “electronic” only if offline products are used. Use “virtual” only in the context of “virtual reality” (3-D worlds). Use “online” only in the context of	Title includes mode of delivery (“mobile digital intervention”), primary condition (“Chronic Kidney Disease”), target group (“CKD patients”), and study design (disclosed as mixed-methods with original RCT registration). No non-web-based co-interventions; title complies with terminology requirements (avoids ambiguous terms).

Section/Topic	Item No.	CONSORT Checklist Item	EHEALTH Extensions (additions to, or clarification of the CONSORT item)	Study Response (Directly Aligned with Manuscript)
			"online support groups". Complement or substitute product names with broader terms for the class of products (such as "mobile" or "smart phone" instead of "iphone"), especially if the application runs on different platforms.ii) Mention non-web-based components or important co-interventions in the title, if any (e.g., "with telephone support").iii) Mention primary condition or target group in the title, if any (e.g., "for children with Type I Diabetes")	
	1b	Structured summary of trial design, methods, results, and conclusions (NPT extension: Description of experimental treatment,	i) Mention key features/functionalities/components of the intervention and comparator in the abstract. If possible, also mention theories and principles used for designing the site. Keep in mind the needs of systematic reviewers and indexers by including important synonyms.ii) Clarify the level of human involvement in the abstract, e.g., use phrases like "fully automated" vs.	Structured abstract (Background-Objectives-Methods-Results-Conclusions) includes:i) Intervention features (gamified modules, personalized reports) and comparator (static educational content) in Su Yi app; theoretical basis (Orem's Self-Care Deficit Nursing Theory).ii) Human involvement: Nurse-led (nurse researchers with master's qualifications, remote monitoring).iii) Recruitment: Offline (from hospital inpatients);

Section/Topic	Item No.	CONSORT Checklist Item	EHEALTH Extensions (additions to, or clarification of the CONSORT item)	Study Response (Directly Aligned with Manuscript)
		comparator, care providers, centers, and blinding status)	“therapist/nurse/care provider/physician-assisted” (mention number and expertise of providers involved, if any).iii) Open vs. closed, web-based (self-assessment) vs. face-to-face assessments in abstract: Mention how participants were recruited (online vs. offline), e.g., from an open access website or from a clinic or a closed online user group (closed usergroup trial), and clarify if this was a purely web-based trial, or there were face-to-face components (as part of the intervention or for assessment). Clearly say if outcomes were self-assessed through questionnaires (as common in web-based trials).iv) Results in abstract must contain use data: Report number of participants enrolled/assessed in each group, the use/uptake of the intervention (e.g., attrition/adherence metrics, use over time, number of logins etc.), in addition to primary/secondary outcomes.v)	face-to-face assessments (outpatient follow-up); outcomes include self-assessed questionnaires (knowledge test, quality of life scales).iv) Use data: 99 enrolled (46 intervention, 53 control); 24% completion rate for 24-hour urine collection; knowledge improvement (P=0.005).v) Conclusions: Negative primary outcome attributed to measurement burden and implementation barriers, not lack of intervention uptake.

Section/Topic	Item No.	CONSORT Checklist Item	EHEALTH Extensions (additions to, or clarification of the CONSORT item)	Study Response (Directly Aligned with Manuscript)
			Conclusions/Discussions in abstract for negative trials: Discuss the primary outcome - if the trial is negative (primary outcome not changed), and the intervention was not used, discuss whether negative results are attributable to lack of uptake and discuss reasons.	
**INTRODUCTION**	2a	Scientific background and explanation of rationale	i) Describe the problem and the type of system/solution that is object of the study: intended as stand-alone intervention vs. incorporated in broader health care program? Intended for a particular patient population? Goals of the intervention, e.g., being more cost-effective to other interventions, replace or complement other solutions?ii) Scientific background, rationale: What is known about the (type of) system that is the object of the study (be sure to discuss the use of similar systems for other conditions/diagnoses, if appropriate),	i) System type: Stand-alone mobile app intervention for CKD patients; goal: complement traditional care to improve sodium restriction adherence.ii) Background: Excessive sodium intake accelerates CKD progression; clinical guidelines recommend restriction but adherence is poor (<30% meet targets). Digital therapeutics show promise but real-world barriers remain. Comparator (minimal digital intervention) chosen to control for digital exposure, enabling evaluation of enhanced features.

Section/Topic	Item No.	CONSORT Checklist Item	EHEALTH Extensions (additions to, or clarification of the CONSORT item)	Study Response (Directly Aligned with Manuscript)
			motivation for the study, i.e., what are the reasons for and what is the context for this specific study, from which stakeholder viewpoint is the study performed, potential impact of findings. Briefly justify the choice of the comparator.	
	2b	Specific objectives or hypotheses	No EHEALTH-specific additions	Objectives: Evaluate a nurse-led digital sodium restriction program via convergent mixed-methods, focusing on implementation processes, patient engagement trajectories, and intervention-lived experience alignment.Hypotheses: 1) The intervention may improve sodium-related knowledge but face high attrition in 24-hour urine collection; 2) Mapping engagement trajectories will identify core implementation barriers.

Section/Topic	Item No.	CONSORT Checklist Item	EHEALTH Extensions (additions to, or clarification of the CONSORT item)	Study Response (Directly Aligned with Manuscript)
**METHODS: Trial Design**	3a	Description of trial design (such as parallel, factorial) including allocation ratio	No EHEALTH-specific additions	Parallel-group RCT with 1:1 allocation ratio; re-conceptualized as exploratory single-center convergent mixed-methods implementation study due to high missing primary outcome data.
	3b	Important changes to methods after trial commencement (such as eligibility criteria), with reasons	i) Bug fixes, Downtimes, Content Changes: ehealth systems are often dynamic systems. A description of changes to methods therefore also includes important changes made on the intervention or comparator during the trial (e.g., major bug fixes or changes in the functionality or content) and other “unexpected events” that may have influenced study design such as staff changes, system failures/downtimes, etc.	Change: Study design adjusted from RCT to mixed-methods implementation study.Reason: 75.8% missing data in primary outcome (24-hour urinary sodium excretion).No bug fixes, downtimes, or content changes to the app during the trial; intervention content was frozen.

Section/Topic	Item No.	CONSORT Checklist Item	EHEALTH Extensions (additions to, or clarification of the CONSORT item)	Study Response (Directly Aligned with Manuscript)
**METHODS: Participants**	4a	Eligibility criteria for participants	i) Computer / Internet literacy is often an implicit “de facto” eligibility criterion - this should be explicitly clarified.ii) Open vs. closed, web-based vs. face-to-face assessments: Mention how participants were recruited (online vs. offline), e.g., from an open access website or from a clinic, and clarify if this was a purely web-based trial, or there were face-to-face components (as part of the intervention or for assessment), i.e., to what degree the study team got to know the participant. In online-only trials, clarify if participants were quasi-anonymous and whether having multiple identities was possible or whether technical or logistical measures (e.g., cookies, email confirmation, phone calls) were used to detect/prevent these.iii) Information given during recruitment. Specify how participants were briefed for recruitment and in the informed consent procedures (e.g., publish the informed consent documentation as	i) Explicit eligibility criterion: Proficiency in using smartphones and mobile applications.ii) Recruitment: Offline (from inpatients admitted to nephrology department); face-to-face components (interviews, outpatient follow-up assessments); participants were known to the study team (not quasi-anonymous); no multiple identity issues (verified via hospital records).iii) Recruitment briefing: Trained nurses explained the study purpose, procedures, and risks; written informed consent obtained (consent documentation not published as appendix but available upon request).

Section/Topic	Item No.	CONSORT Checklist Item	EHEALTH Extensions (additions to, or clarification of the CONSORT item)	Study Response (Directly Aligned with Manuscript)
			appendix), as this information may have an effect on user self-selection, user expectation and may also bias results.	
	4b	Settings and locations where the data were collected	i) Clearly report if outcomes were (self-)assessed through online questionnaires (as common in web-based trials) or otherwise.ii) Report how institutional affiliations are displayed to potential participants [on ehealth media], as affiliations with prestigious hospitals or universities may affect volunteer rates, use, and reactions with regards to an intervention. (Not a required item – describe only if this may bias results)	i) Outcomes assessed via: Online app logging (blood pressure), face-to-face measurements (laboratory parameters, blood pressure), and paper/electronic questionnaires (knowledge test, quality of life).ii) Institutional affiliation (Army Medical University) was disclosed to participants during recruitment; no evidence of bias from this disclosure.
**METHODS: Interventions**	5	The interventions for each group with sufficient details to allow	i) Mention names, credential, affiliations of the developers, sponsors, and owners (if authors/evaluators are owners or developer of the software, this needs to be declared in a "Conflict of interest" section or mentioned	i) Developers: Nurse researchers from Department of Nephrology, Xinqiao Hospital, Army Medical University; Sponsor: Chongqing Municipal Science-Technology Bureau and Health Commission; No conflict of interest

Section/Topic	Item No.	CONSORT Checklist Item	EHEALTH Extensions (additions to, or clarification of the CONSORT item)	Study Response (Directly Aligned with Manuscript)
		replication, including how and when they were actually administered	elsewhere in the manuscript).ii) Describe the history/development process of the application and previous formative evaluations (e.g., focus groups, usability testing), as these will have an impact on adoption/use rates and help with interpreting results.iii) Revisions and updating. Clearly mention the date and/or version number of the application/intervention (and comparator, if applicable) evaluated, or describe whether the intervention underwent major changes during the evaluation process, or whether the development and/or content was "frozen" during the trial. Describe dynamic components such as news feeds or changing content which may have an impact on the replicability of the intervention.iv) Provide information on quality assurance methods to ensure accuracy and quality of information provided, if applicable.v) Ensure replicability by publishing the source code,	(authors are not owners of the software).ii) Development process: Based on Orem's Self-Care Deficit Nursing Theory and CKD sodium restriction guidelines; pilot tested with 2 participants to refine the interview guide (no formal usability testing of the app).iii) Version: No version number; content frozen during the trial; no dynamic components (e.g., news feeds).iv) Quality assurance: Educational content reviewed by 3 nephrology experts; questionnaire validated (content validity index=0.92, Cronbach's α=0.78).v) Replicability: Flowcharts of the intervention process provided (Figure 1); screenshots included in the manuscript; source code not published.vi) Digital preservation: No URL provided; screenshots archived as part of the manuscript supplementary materials.vii) Access: Participants accessed the app via personal smartphones; no payment required; no membership restrictions; demo mode available for reviewers upon request.viii) Mode of

Section/Topic	Item No.	CONSORT Checklist Item	EHEALTH Extensions (additions to, or clarification of the CONSORT item)	Study Response (Directly Aligned with Manuscript)
			and/or providing screenshots/screen-capture video, and/or providing flowcharts of the algorithms used.vi) Digital preservation: Provide the URL of the application, but as the intervention is likely to change or disappear over the course of the years; also make sure the intervention is archived (Internet Archive, webcitation.org, and/or publishing the source code or screenshots/videos alongside the article). As pages behind login screens cannot be archived, consider creating demo pages which are accessible without login.vii) Access: Describe how participants accessed the application, in what setting/context, if they had to pay (or were paid) or not, whether they had to be a member of specific group. If known, describe how participants obtained "access to the platform and Internet". To ensure access for editors/reviewers/readers, consider to provide a "backdoor" login account or demo	delivery: Mobile app-based; Intervention group features: gamified learning modules, in-app quizzes, peer competition, behavioral practice tasks, personalized reports (tailored to knowledge mastery and sodium intake trends); Comparator features: static video and text content, blood pressure logging; Theoretical framework: Orem's Self-Care Deficit Nursing Theory + Empowerment Theory; Content source: CKD sodium restriction guidelines and nursing expertise; Communication: Asynchronous (app-based, no real-time communication).ix) Use parameters: Intervention group: 4-week program (1 knowledge point/day, quizzes after each knowledge point, weekly competition); Control group: Daily blood pressure logging; Use ad libitum beyond required tasks.x) Human involvement: Nurse-led remote monitoring (no regular synchronous support); 2 nurse researchers (master's qualifications) conducted interviews; No technical assistance provided

Section/Topic	Item No.	CONSORT Checklist Item	EHEALTH Extensions (additions to, or clarification of the CONSORT item)	Study Response (Directly Aligned with Manuscript)
			mode for reviewers/readers to explore the application.viii) Describe mode of delivery, features/functionalities/components of the intervention and comparator, and the theoretical framework used to design them (instructional strategy, behaviour change techniques, persuasive features, etc.). This includes an in-depth description of the content (including where it is coming from and who developed it), "whether [and how] it is tailored to individual circumstances and allows users to track their progress and receive feedback". This also includes a description of communication delivery channels and – if computer-mediated communication is a component – whether communication was synchronous or asynchronous. It also includes information on presentation strategies, including page design principles, average amount of text on pages, presence of hyperlinks to other resources etc.ix) Describe use parameters	during the trial; Routine application would require minimal human involvement.xi) Prompts/reminders: Control group received daily app prompts to log blood pressure; Intervention group received app prompts for quizzes and tasks; No additional prompts (e.g., SMS, phone calls); Routine application would retain app-based prompts.xii) Co-interventions: Single educational session on sodium restriction for both groups; No additional training/support; Routine application would not require the educational session.

Section/Topic	Item No.	CONSORT Checklist Item	EHEALTH Extensions (additions to, or clarification of the CONSORT item)	Study Response (Directly Aligned with Manuscript)
			(e.g., intended “doses” and optimal timing for use). Clarify what instructions or recommendations were given to the user, e.g., regarding timing, frequency, heaviness of use, if any, or was the intervention used ad libitum.x) Clarify the level of human involvement (care providers or health professionals, also technical assistance) in the e-intervention or as co-intervention. Detail number and expertise of professionals involved, if any, as well as “type of assistance offered, the timing and frequency of the support, how it is initiated, and the medium by which the assistance is delivered”. It may be necessary to distinguish between the level of human involvement required for the trial, and the level of human involvement required for a routine application outside of a RCT setting.xi) Report any prompts/reminders used: Clarify if there were prompts (letters, emails, phone calls, SMS) to use the	

Section/Topic	Item No.	CONSORT Checklist Item	EHEALTH Extensions (additions to, or clarification of the CONSORT item)	Study Response (Directly Aligned with Manuscript)
			application, what triggered them, frequency, etc. It may be necessary to distinguish between the level of prompts/reminders required for the trial, and the level of prompts/reminders for a routine application outside of a RCT setting.xii) Describe any co-interventions (incl. training/support): Clearly state any "interventions that are provided in addition to the targeted eHealth intervention", as ehealth intervention may not be designed as standalone intervention. This includes training sessions and support. It may be necessary to distinguish between the level of training required for the trial, and the level of training for a routine application outside of a RCT setting.	

Section/Topic	Item No.	CONSORT Checklist Item	EHEALTH Extensions (additions to, or clarification of the CONSORT item)	Study Response (Directly Aligned with Manuscript)
**METHODS: Outcomes**	6a	Completely defined pre-specified primary and secondary outcome measures, including how and when they were assessed	i) If outcomes were obtained through online questionnaires, describe if they were validated for online use and apply CHERRIES items to describe how the questionnaires were designed/deployed.ii) Describe whether and how "use" (including intensity of use/dosage) was defined/measured/monitored (logins, logfile analysis, etc.). Use/adoption metrics are important process outcomes that should be reported in any ehealth trial.iii) Describe whether, how, and when qualitative feedback was obtained from participants (e.g., through emails, feedback forms, interviews, focus groups).	i) Online questionnaires: Knowledge test (validated for offline use, not specifically for online; CHERRIES items applied: anonymous responses, clear instructions).ii) Use measurement: Monitored via app logins, task completion rates, and 24-hour urine collection completion rate (24.2%); No logfile analysis of session length/frequency.iii) Qualitative feedback: Obtained via semi-structured in-depth interviews (20-40 minutes) with 23 intervention completers after 3-month follow-up.
	6b	Any changes to trial outcomes after the trial commenced, with reasons	No EHEALTH-specific additions	No changes to pre-specified outcomes; Primary outcome (24-hour urinary sodium excretion) was downgraded to exploratory status due to high missingness.

Section/Topic	Item No.	CONSORT Checklist Item	EHEALTH Extensions (additions to, or clarification of the CONSORT item)	Study Response (Directly Aligned with Manuscript)
**METHODS: Sample Size**	7a	How sample size was determined (NPT: When applicable, details of whether and how the clustering by care provides or centers was addressed)	i) Describe whether and how expected attrition was taken into account when calculating the sample size	Calculated using PASS 15.0 software; Assumptions: Between-group difference of 26 mmol/day in 24-hour urinary sodium excretion, SD=34.0, α=0.05, 90% power; 37 participants per group required; Expected attrition of 20% considered, target sample size 47 per group (total n=94); Actual enrollment 99 (46 intervention, 53 control).
	7b	When applicable, explanation of any interim analyses and stopping guidelines	No EHEALTH-specific additions	No interim analyses or stopping guidelines pre-specified.

Section/Topic	Item No.	CONSORT Checklist Item	EHEALTH Extensions (additions to, or clarification of the CONSORT item)	Study Response (Directly Aligned with Manuscript)
**METHODS: Randomisation: Sequence Generation**	8a	Method used to generate the random allocation sequence (NPT: When applicable, how care providers were allocated to each trial group)	No EHEALTH-specific additions	Computer-generated random sequences in SPSS 24.0; No care provider allocation (single center, uniform care providers).
	8b	Type of randomisation; details of any restriction (such as blocking and block size)	No EHEALTH-specific additions	Simple randomization; No blocking or restrictions.

Section/Topic	Item No.	CONSORT Checklist Item	EHEALTH Extensions (additions to, or clarification of the CONSORT item)	Study Response (Directly Aligned with Manuscript)
**METHODS: Allocation Concealment Mechanism**	9	Mechanism used to implement the random allocation sequence (such as sequentially numbered containers), describing any steps taken to conceal the sequence until interventions were assigned	No EHEALTH-specific additions	Sequentially numbered, opaque sealed envelopes prepared by an independent statistician; Concealed until participant enrollment and assignment.

Section/Topic	Item No.	CONSORT Checklist Item	EHEALTH Extensions (additions to, or clarification of the CONSORT item)	Study Response (Directly Aligned with Manuscript)
**METHODS: Implementation**	10	Who generated the random allocation sequence, who enrolled participants, and who assigned participants to interventions	No EHEALTH-specific additions	Random sequence generated by an independent statistician; Participants enrolled by trained clinical nurses; Assignments made by nurses via opening sealed envelopes.
**METHODS: Blinding**	11a	If done, who was blinded after assignment to interventions (for example, participants, care providers, those assessing outcomes) and how (NPT:	i) Specify who was blinded, and who wasn't. Usually, in web-based trials it is not possible to blind the participants (this should be clearly acknowledged), but it may be possible to blind outcome assessors, those doing data analysis or those administering co-interventions (if any).ii) Informed consent procedures (4a-ii) can create biases and certain expectations - discuss e.g., whether participants knew which	i) Blinded: Outcome assessors, laboratory technicians, data analysts; Unblinded: Participants (different app functionalities), intervention delivery nurses; Blinding maintained via anonymous outcome data collection.ii) Participants were informed of two study groups (enhanced vs. minimal digital intervention); No evidence of expectation bias.

Section/Topic	Item No.	CONSORT Checklist Item	EHEALTH Extensions (additions to, or clarification of the CONSORT item)	Study Response (Directly Aligned with Manuscript)
		Whether or not administering co-interventions were blinded to group assignment)	intervention was the "intervention of interest" and which one was the "comparator".	
	11b	If relevant, description of the similarity of interventions	No EHEALTH-specific additions	Interventions similar in delivery mode (Su Yi app) but differ in content/features; No sham intervention possible due to distinct functionalities.
**METHODS: Statistical Methods**	12a	Statistical methods used to compare groups for primary and secondary outcomes (NPT: When applicable, details of	i) Imputation techniques to deal with attrition / missing values: Not all participants will use the intervention/comparator as intended and attrition is typically high in ehealth trials. Specify how participants who did not use the application or dropped out from the trial were treated in the statistical analysis (a complete case analysis is strongly	i) Missing data handling: ITT analysis for available data; No between-group comparisons for primary outcome (75.8% missing); No multiple imputation (untestable assumptions); Complete case analysis for secondary outcomes.Statistical tests: Shapiro-Wilk normality test, independent samples t-test, Mann-Whitney U test, chi-square test; SAS version 9.4 used.

Section/Topic	Item No.	CONSORT Checklist Item	EHEALTH Extensions (additions to, or clarification of the CONSORT item)	Study Response (Directly Aligned with Manuscript)
		whether and how the clustering by care providers or centers was addressed)	discouraged, and simple imputation techniques such as LOCF may also be problematic).	
	12b	Methods for additional analyses, such as subgroup analyses and adjusted analyses	No EHEALTH-specific additions	No subgroup analyses or adjusted analyses pre-specified; No additional analyses conducted.This is because the research design was changed during the study process
**METHODS: Ethics & Informed Consent (X26)**	-	-	i) Comment on ethics committee approval.ii) Outline informed consent procedures e.g., if consent was obtained offline or online (how? Checkbox, etc.?), and what information was provided.iii) Safety and security procedures, incl. privacy considerations, and “any steps taken to reduce the likelihood or detection of harm	i) Ethics approval: Institutional Review Board of the Second Affiliated Hospital of Army Medical University (Approval No. 2022-Research-040-01; revised design approval No. 2022-Research-515-01).ii) Informed consent: Obtained offline (written signature) after detailed verbal explanation; Information provided included study purpose, procedures,

Section/Topic	Item No.	CONSORT Checklist Item	EHEALTH Extensions (additions to, or clarification of the CONSORT item)	Study Response (Directly Aligned with Manuscript)
			(e.g., education and training, availability of a hotline)”.	risks, benefits, and data anonymization.iii) Safety/security: Data anonymization (interview transcripts labeled P1-P23); Confidential storage of audio recordings and data; No hotline provided (participants instructed to contact their attending physician for health concerns).
**RESULTS: Participant Flow**	13a	For each group, the numbers of participants who were randomly assigned, received intended treatment, and were analysed for the primary outcome (NPT: The number of care providers or centers	No EHEALTH-specific additions	Randomly assigned: 46 (intervention), 53 (control); Received intended treatment: 45 (intervention, 1 death), 52 (control, 1 withdrawal); Analysed for primary outcome: 11 (intervention), 13 (control); 1 center, no care provider clustering.

Section/Topic	Item No.	CONSORT Checklist Item	EHEALTH Extensions (additions to, or clarification of the CONSORT item)	Study Response (Directly Aligned with Manuscript)
		performing the intervention in each group and the number of patients treated by each care provider in each center)		
	13b	For each group, losses and exclusions after randomisation, together with reasons	i) Strongly recommended: An attrition diagram (e.g., proportion of participants still logging in or using the intervention/comparator in each group plotted over time, similar to a survival curve) or other figures or tables demonstrating usage/dose/engagement.	Losses: 1 death (intervention, unrelated to study), 1 withdrawal (control, personal reasons); Exclusions: None after randomization.Attrition diagram provided (Figure 2); Usage demonstrated via completion rates (24-hour urine collection: 23.9% intervention, 24.5% control).
**RESULTS: Recruitment**	14a	Dates defining the periods of recruitment and follow-up	i) Indicate if critical "secular events" fell into the study period, e.g., significant changes in Internet resources available or "changes in computer hardware or Internet delivery resources".	Recruitment period: June 2022 - October 2022; Follow-up period: 3 months post-intervention.No critical secular events during the study period.

Section/Topic	Item No.	CONSORT Checklist Item	EHEALTH Extensions (additions to, or clarification of the CONSORT item)	Study Response (Directly Aligned with Manuscript)
	14b	Why the trial ended or was stopped [early]	No EHEALTH-specific additions	Trial ended as planned after completing recruitment and follow-up; No early stopping.
**RESULTS: Baseline Data**	15	A table showing baseline demographic and clinical characteristics for each group (NPT: When applicable, a description of care providers (case volume, qualification, expertise, etc.) and centers (volume) in each group)	i) In ehealth trials it is particularly important to report demographics associated with digital divide issues, such as age, education, gender, social-economic status, computer/Internet/ehealth literacy of the participants, if known.	Baseline characteristics table provided (Table 1); Demographics reported: age (median 44-48 years), gender (47.83-60.38% male), education (primary to bachelor’s or above), social-economic status (profession), CKD stage (1-5); Computer/Internet literacy: All participants proficient (inclusion criterion), no additional details reported.

Section/Topic	Item No.	CONSORT Checklist Item	EHEALTH Extensions (additions to, or clarification of the CONSORT item)	Study Response (Directly Aligned with Manuscript)
**RESULTS: Numbers Analysed**	16	For each group, number of participants (denominator) included in each analysis and whether the analysis was by original assigned groups	i) Report multiple "denominators" and provide definitions: Report N's (and effect sizes) "across a range of study participation [and use] thresholds", e.g., N exposed, N consented, N used more than x times, N used more than y weeks, N participants "used" the intervention/comparator at specific pre-defined time points of interest (in absolute and relative numbers per group). Always clearly define "use" of the intervention.ii) Primary analysis should be intent-to-treat; secondary analyses could include comparing only "users", with the appropriate caveats that this is no longer a randomized sample.	Analyses by original assigned groups (ITT); Denominators reported for each outcome:- Sodium knowledge: 40 (intervention), 50 (control);- Quality of life: 40 (intervention), 49 (control);- 24-hour urine collection: 11 (intervention), 13 (control)."Use" defined as completion of assigned app tasks; No secondary analyses of "users only" due to small sample size.

Section/Topic	Item No.	CONSORT Checklist Item	EHEALTH Extensions (additions to, or clarification of the CONSORT item)	Study Response (Directly Aligned with Manuscript)
**RESULTS: Outcomes and Estimation**	17a	For each primary and secondary outcome, results for each group, and the estimated effect size and its precision (such as 95% confidence interval)	i) In addition to primary/secondary (clinical) outcomes, the presentation of process outcomes such as metrics of use and intensity of use (dose, exposure) and their operational definitions is critical. This does not only refer to metrics of attrition (13-b) (often a binary variable), but also to more continuous exposure metrics such as "average session length". These must be accompanied by a technical description how a metric like a “session” is defined (e.g., timeout after idle time).	Primary outcome: No between-group comparison (high missingness); Completers’ median sodium excretion: 97.30 mmol/day (intervention), 154.44 mmol/day (control).Secondary outcome: Sodium knowledge (intervention median 8.00 vs. control 6.00, p=0.005, rank-biserial correlation r=0.32, 95% CI:0.10-0.51).Process outcomes: 24-hour urine collection completion rate (24.2%), questionnaire completion rates (81.8-94.3%); No continuous exposure metrics (e.g., session length) reported.
	17b	For binary outcomes, presentation of both absolute and relative effect sizes is recommended	No EHEALTH-specific additions	No binary primary outcomes; Secondary binary outcomes not reported.

Section/Topic	Item No.	CONSORT Checklist Item	EHEALTH Extensions (additions to, or clarification of the CONSORT item)	Study Response (Directly Aligned with Manuscript)
**RESULTS: Ancillary Analyses**	18	Results of any other analyses performed, including subgroup analyses and adjusted analyses, distinguishing pre-specified from exploratory	i) A subgroup analysis of comparing only users is not uncommon in ehealth trials, but if done it must be stressed that this is a self-selected sample and no longer an unbiased sample from a randomized trial.	No subgroup analyses or adjusted analyses performed.
**RESULTS: Harms**	19	All important harms or unintended effects in each group (for specific guidance see CONSORT for harms)	i) Include privacy breaches, technical problems. This does not only include physical “harm” to participants, but also incidents such as perceived or real privacy breaches, technical problems, and other unexpected/unintended incidents. “Unintended effects” also includes unintended positive effects.ii) Include qualitative feedback from participants or	i) No harms, privacy breaches, or technical problems reported; 1 death (intervention) and 1 withdrawal (control) unrelated to the intervention.ii) Qualitative feedback: Strengths (knowledge improvement, personalized reports); Shortcomings (measurement burden, digital fatigue, social resistance); Reasons for non-use: Urine collection inconvenience, app content monotony.

Section/Topic	Item No.	CONSORT Checklist Item	EHEALTH Extensions (additions to, or clarification of the CONSORT item)	Study Response (Directly Aligned with Manuscript)
			observations from staff/researchers, if available, on strengths and shortcomings of the application, especially if they point to unintended/unexpected effects or uses. This includes (if available) reasons for why people did or did not use the application as intended by the developers.	
**DISCUSSION: Interpretation/Principal Findings**	22	Interpretation consistent with results, balancing benefits and harms, and considering other relevant evidence (NPT: In addition, take into account the choice of the comparator, lack of or partial blinding,	i) Restate study questions and summarize the answers suggested by the data, starting with primary outcomes and process outcomes (use).ii) Highlight unanswered new questions, suggest future research.	i) Study questions restated and answered: Intervention improves sodium knowledge (secondary outcome) but fails to show behavioral/physiological effects (primary outcome); Process outcomes reveal implementation barriers (measurement burden, digital fatigue, social resistance).ii) Unanswered questions: How to optimize outcome measures for real-world feasibility; How to design stage-matched interventions for sustained engagement; Future research: Multi-center pragmatic trials, inclusion of non-completers, evaluation of AI-based monitoring tools.

Section/Topic	Item No.	CONSORT Checklist Item	EHEALTH Extensions (additions to, or clarification of the CONSORT item)	Study Response (Directly Aligned with Manuscript)
		and unequal expertise of care providers or centers in each group)		
**DISCUSSION: Limitations**	20	Trial limitations, addressing sources of potential bias, imprecision, and, if relevant, multiplicity of analyses	i) Typical limitations in ehealth trials: Participants in ehealth trials are rarely blinded. Ehealth trials often look at a multiplicity of outcomes, increasing risk for a Type I error. Discuss biases due to non-use of the intervention/usability issues, biases through informed consent procedures, unexpected events.	Limitations discussed:- Blinding: Participants and nurses unblinded, potential performance bias.- Non-use: Low completion rate of primary outcome (measurement burden), selection bias in qualitative sample (only completers).- Usability: No formal usability testing, potential usability barriers not captured.- Multiplicity of outcomes: No Type I error adjustment, but only one significant secondary outcome.- Other: Single-center design, no subgroup analyses.

Section/Topic	Item No.	CONSORT Checklist Item	EHEALTH Extensions (additions to, or clarification of the CONSORT item)	Study Response (Directly Aligned with Manuscript)
**DISCUSSION: Generalisability**	21	Generalisability (external validity, applicability) of the trial findings (NPT: External validity of the trial findings according to the intervention, comparators, patients, and care providers or centers involved in the trial)	i) Generalizability to other populations: In particular, discuss generalizability to a general Internet population, outside of a RCT setting, and general patient population, including applicability of the study results for other organizations.ii) Discuss if there were elements in the RCT that would be different in a routine application setting (e.g., prompts/reminders, more human involvement, training sessions or other co-interventions) and what impact the omission of these elements could have on use, adoption, or outcomes if the intervention is applied outside of a RCT setting.	i) Generalizability: Applicable to smartphone-proficient CKD patients in tertiary hospitals; Limited to Chinese population, non-smartphone users, and primary care settings; Implementation barriers (measurement burden, digital fatigue) transferable to similar chronic disease digital interventions.ii) RCT vs. routine application: Routine setting would omit the single educational session; Retain app-based prompts; Require minimal human involvement; Omission of educational session may reduce initial engagement but improve scalability.

Section/Topic	Item No.	CONSORT Checklist Item	EHEALTH Extensions (additions to, or clarification of the CONSORT item)	Study Response (Directly Aligned with Manuscript)
**OTHER INFORMATION: Registration**	23	Registration number and name of trial registry	No EHEALTH-specific additions	Trial registry: Chinese Clinical Trial Registry; Registration number: ChiCTR2200060627; Registered on 06 June 2022 (prospective).
**OTHER INFORMATION: Protocol**	24	Where the full trial protocol can be accessed, if available	No EHEALTH-specific additions	The full trial protocol can be accessed on the Chinese Clinical Trial Registry website.
**OTHER INFORMATION: Funding**	25	Sources of funding and other support (such as supply of drugs), role of funders	No EHEALTH-specific additions	Funding source: Chongqing Municipal Science-Technology Bureau and Health Commission jointly funded project (grant number 2022MSXM026); The first author serves as the principal investigator of this funded project and oversees the whole research process. No external commercial funders are involved in this study, and no conflicts of interest exist among all authors.
**OTHER INFORMATION: Competing Interests**	-	-	i) In addition to the usual declaration of interests (financial or otherwise), also state the “relation of the study team towards the	Authors declare no financial or non-financial competing interests; Study team (nurse researchers) are distinct from the app developer

Section/Topic	Item No.	CONSORT Checklist Item	EHEALTH Extensions (additions to, or clarification of the CONSORT item)	Study Response (Directly Aligned with Manuscript)
**(X27)**			system being evaluated", i.e., state if the authors/evaluators are distinct from or identical with the developers/sponsors of the intervention.	(Senmei Company, Chongqing); No relation to the system beyond research evaluation.
