# Supplementary material for: Nurse-Led Digital Intervention for Sodium Restriction in Chronic Kidney Disease: Mixed Methods Implementation Study
Source: JMIR Nurs. 2026 Jul 20;9:e94330. doi: 10.2196/94330 (PMC13384475; doi:10.2196/94330)
Supplement: Checklist 2 [file nursing-v9-e94330-s004.pdf]

COREQ Checklist for Qualitative Research

Manuscript Title: Nurse-Led Mobile Digital Intervention for Sodium Restriction in Chronic Kidney Disease: An Exploratory Mixed-Methods Study on Patient Journey and Implementation Barriers

Manuscript Number: 94330

Corresponding Author: Jinghong Zhao

Date Completed: 2026.04.08

Qualitative Methodology: Semi-structured in-depth interviews + Thematic analysis (hybrid deductive-inductive approach)

Item No.	COREQ Core Domains	Your Study Response (Aligned with Manuscript Content)	Manuscript Citation/Section
**1. Research Team & Reflexivity**	1.1 Interviewer/facilitator characteristics (professional background, training)	Two nurse researchers with master's qualifications; specialized training in qualitative research methods (thematic analysis, semi-structured interview skills); no prior relationship with participants	Methods 2.6
	1.2 Interviewer/facilitator characteristics (gender, age, ethnicity)	Gender: Mixed (1 male, 1 female); Age: 30-35 years; Ethnicity: Han Chinese (consistent with participants' ethnicity)	Methods 2.6 (supplemented per COREQ)

Item No.	COREQ Core Domains	Your Study Response (Aligned with Manuscript Content)	Manuscript Citation/Section
	1.3 Reflexivity (researchers' assumptions, biases, and how they were addressed)	Researchers' initial assumption: Digital interventions' adherence barriers are mainly technical; addressed via regular debriefing sessions during data collection to enhance reflexivity, and peer review of coding results to minimize subjective bias	Methods 2.6
**2. Study Design**	2.1 Theoretical framework	Guided by Orem's Self-Care Deficit Nursing Theory (core) integrated with Empowerment Theory; framework informed interview guide development and thematic analysis	Methods 2.6; Discussion 4.3
	2.2 Rationale for qualitative approach	To explore in-depth participants' dynamic engagement experiences, emotional shifts, and implementation barriers, which quantitative data cannot fully capture	Introduction 3; Methods 2.6
	2.3 Research question(s)	What are the dynamic engagement experiences, encountered barriers, and evolving needs of CKD patients during the nurse-led digital sodium restriction intervention?	Introduction 4; GRAMMS Checklist
	2.4 Sampling strategy	Purposive sampling; inclusion criteria: Completed the full 4-week digital intervention, willing to participate in interviews, able to express subjective experiences clearly	Methods 2.6

Item No.	COREQ Core Domains	Your Study Response (Aligned with Manuscript Content)	Manuscript Citation/Section
	2.5 Sample size	23 participants; data saturation achieved after 18 interviews (subsequent 5 interviews yielded no new themes)	Results 3.3
	2.6 Recruitment method	Recruited from intervention group completers; invited by trained clinical nurses during 3-month follow-up visits; verbal and written informed consent obtained	Methods 2.6; Results 3.1
**3. Data Collection**	3.1 Data collection method	Semi-structured in-depth interviews; face-to-face format; conducted in Mandarin Chinese	Methods 2.6
	3.2 Interview guide (development, pilot testing)	Developed based on theoretical framework and research questions; included 5 modules (warm-up, initial contact, sustained interaction, relationship evolution, summary); pilot tested with 2 participants (revised ambiguous questions, e.g., clarified "platform operation difficulties" to specific function-related queries)	Methods 2.6; Supplementary Material 2
	3.3 Interview duration	20-40 minutes per session (median: 30 minutes)	Methods 2.6
	3.4 Interview setting	Outpatient CKD management center; private consultation room to ensure confidentiality and minimize distractions	Methods 2.6
	3.5 Recording method	Audio-recorded using professional recording devices; participants consented to recording	Methods 2.6

Item No.	COREQ Core Domains	Your Study Response (Aligned with Manuscript Content)	Manuscript Citation/Section
	3.6 Field notes	Researchers took brief field notes during interviews (e.g., participants' non-verbal behaviors: hesitation, excitement); integrated into transcript analysis	Methods 2.6
	3.7 Transcription process	Verbatim transcription by professional transcriptionists; anonymized (labeled as P1-P23); transcripts reviewed by interviewers for accuracy	Methods 2.6
	3.8 Translation (if applicable)	Transcripts translated from Mandarin to English via double translation + back-translation; translators with master's degree in medical English and experience in qualitative research translation; fidelity verified by research team	Methods 2.6
**4. Data Analysis**	4.1 Data analysis method	Thematic analysis; hybrid deductive-inductive approach (initial coding based on predefined domains: tasks, emotions, pain points, needs; emergent themes refined iteratively)	Methods 2.7.2
	4.2 Software used for analysis	NVivo 12 (QSR International) for data management and coding	Methods 2.7.2
	4.3 Coding process	Two researchers independently coded 20% of transcripts; inter-coder reliability tested (Cohen's kappa=0.72); discrepancies resolved via group discussion; finalized codebook applied to full dataset	Methods 2.6; 2.7.2

Item No.	COREQ Core Domains	Your Study Response (Aligned with Manuscript Content)	Manuscript Citation/Section
	4.4 Theme development	Themes derived from coding results; aligned with theoretical framework and research questions; validated by the research team (including 1 qualitative research expert)	Results 3.3; Supplementary Material 2
**5. Reporting**	5.1 Participant characteristics (demographics)	Reported in Table 3: Gender (11 male, 12 female), age (20-61 years), educational level (primary to bachelor's or above), profession, CKD stage (1-5)	Results 3.3 (Table 3)
	5.2 Ethical approval	Approved by the Institutional Review Board of the Second Affiliated Hospital of Army Medical University (Approval No. 2022-Research-040-01); revised study design approved (No. 2022-Research-515-01)	Methods 2.1
	5.3 Participants' consent	Written informed consent obtained before interviews; consent for audio recording and data use in research/publication	Methods 2.6
	5.4 Anonymization	Participants anonymized (P1-P23); no personal identifying information (e.g., name, medical record number) in transcripts or quotes	Methods 2.6; Results 3.3
	5.5 Quotations (use of participant quotes to support themes)	Key themes supported by anonymized participant quotes (e.g., "Urine collection is too troublesome, I can't do it while working" — P4)	Results 3.3; 3.4

Item No.	COREQ Core Domains	Your Study Response (Aligned with Manuscript Content)	Manuscript Citation/Section
	5.6 Rigor (strategies to enhance trustworthiness)	- Credibility: Member checking (10 participants reviewed transcript excerpts and theme summaries for accuracy); - Dependability: Detailed coding manual and process documentation; - Transferability: Comprehensive reporting of participant characteristics and context; - Confirmability: Independent coding and consensus-based discrepancy resolution	Methods 2.6; 2.7.2
