# Supplementary material for: Nurse-Led Digital Intervention for Sodium Restriction in Chronic Kidney Disease: Mixed Methods Implementation Study
Source: JMIR Nurs. 2026 Jul 20;9:e94330. doi: 10.2196/94330 (PMC13384475; doi:10.2196/94330)
Supplement: Checklist 3 [file nursing-v9-e94330-s005.pdf]

GRAMMS Checklist for Mixed Methods Studies

Manuscript Title: Nurse-Led Mobile Digital Intervention for Sodium Restriction in Chronic Kidney Disease: An Exploratory Mixed-Methods Study on Patient Journey and Implementation Barriers

Manuscript Number: 94330

Corresponding Author: Jinghong Zhao

Date Completed: 2026.04.08

No.	GRAMMS Core Items	Specific Description	Your Manuscript Response (Fill in Based on Actual Content)
1	Study Design Identification	Clearly state the mixed methods design type (e.g., convergent parallel, explanatory sequential) and rationale	Convergent parallel mixed methods design was adopted. Quantitative and qualitative data were collected in parallel, analyzed separately, and integrated through a joint display matrix to comprehensively explore implementation barriers and patient engagement experiences of the nurse-led digital sodium restriction intervention.
2	Theoretical Framework	Specify the theoretical basis guiding the study design and integration of quantitative/qualitative components	The study was guided by Orem's Self-Care Deficit Nursing Theory (core framework) integrated with the core concepts of Empowerment Theory. The theoretical framework informed intervention design, data collection (e.g., interview guide development), and results integration.
3	Research Questions	Present mixed methods research questions, including quantitative, qualitative, and integrated questions	- Quantitative question: Does the nurse-led digital sodium restriction intervention improve sodium-related knowledge, 24-hour urinary sodium excretion, blood pressure, and quality of life in CKD patients?- Qualitative question: What are the dynamic engagement experiences, barriers, and needs of

No.	GRAMMS Core Items	Specific Description	Your Manuscript Response (Fill in Based on Actual Content)
			CKD patients during the digital sodium restriction intervention?- Integrated question: How do quantitative outcomes (e.g., knowledge improvement vs. low urine collection completion) align with qualitative findings to explain the knowledge-behavior gap in digital sodium restriction interventions?
4	Quantitative Methods	Detail quantitative sampling strategy, sample size, data collection tools, and statistical analysis methods	- Sampling: 1:1 randomized sampling from inpatients with CKD stages 1-5 in the nephrology department- Sample size: 99 participants (46 in intervention group, 53 in control group), calculated using PASS 15.0 software- Tools: 24-hour urinary sodium excretion measurement, self-developed 10-item sodium control knowledge questionnaire (Cronbach's α=0.78, CVI=0.92), KDQOL-SF™ 1.3, blood pressure and laboratory parameter testing- Analysis: Shapiro-Wilk normality test, independent samples t-test, Mann-Whitney U test, chi-square test, ITT analysis (SAS 9.4)
5	Qualitative Methods	Describe qualitative sampling strategy, sample size, data collection approaches, and analytical methods	- Sampling: Purposive sampling of 23 intervention completers- Data collection: Semi-structured in-depth interviews (20-40 minutes per session, Mandarin), audio-recorded, verbatim transcribed, anonymized, and double translation + back-translation for English transcripts- Analysis: Thematic analysis (NVivo 12), hybrid deductive-inductive coding, inter-coder reliability testing (Cohen's kappa=0.72)
6	Integration Strategy	Explain when (timing) and how (method) quantitative and qualitative data were integrated	- Timing: Integration occurred after separate quantitative and qualitative analysis (post-analysis integration)- Method: Joint display matrix (Table 4) with columns for quantitative outcomes, qualitative themes, integration types (convergence, divergence, expansion), and interpretations

No.	GRAMMS Core Items	Specific Description	Your Manuscript Response (Fill in Based on Actual Content)
7	Quantitative Results	Report key quantitative findings, including descriptive and inferential statistics (effect sizes, confidence intervals)	- Sodium control knowledge: Intervention group median 8.00 (7.00-8.00) vs. control group 6.00 (6.00-8.00), p=0.005, rank-biserial correlation r=0.32 (95% CI:0.10-0.51)- 24-hour urinary sodium collection completion rate: 24.2% (11 in intervention group, 13 in control group)- No significant differences in blood pressure, quality of life (SF-36, KDTA), or laboratory parameters between groups
8	Qualitative Results	Present key qualitative themes with supporting participant quotes	- Core themes: Three-phase patient journey (Initial Contact and Expectation, Sustained Interaction and Empowerment, Platform-Facilitated Relationship Evolution)- Key themes: Measurement burden, digital fatigue, social resistance, active self-monitoring- Example quote: "Urine collection is too troublesome, I can't do it while working" (P4)
9	Integrated Results	Report integrated findings, including convergence, divergence, or expansion of quantitative and qualitative data	- Convergence: Quantitative knowledge improvement aligned with qualitative theme of "active knowledge acquisition"- Divergence: High knowledge gain (quantitative) contrasted with low urine collection completion (quantitative) and qualitative theme of "measurement burden," revealing the "measurement paradox"- Expansion: Qualitative theme of "social resistance" explained the lack of between-group difference in blood pressure (quantitative)
10	Discussion	Interpret mixed methods findings holistically, address research questions, and connect to the theoretical framework	- Holistic interpretation: Nurse-led digital interventions improve sodium knowledge but fail to translate into behavioral change due to implementation barriers (measurement burden, digital fatigue, social resistance)- Theoretical connection: Findings align with Orem's Self-Care Deficit Nursing Theory (self-care requires knowledge, skills, and environmental support) and

No.	GRAMMS Core Items	Specific Description	Your Manuscript Response (Fill in Based on Actual Content)
			Empowerment Theory (motivation and autonomy are key to sustained engagement)- Research question response: The knowledge-behavior gap is attributed to misalignment between intervention demands and patients' daily realities
11	Limitations	Acknowledge limitations of both quantitative and qualitative components, and mixed methods integration	- Quantitative limitations: High missing data (75.8%) for 24-hour urinary sodium excretion, single-center design- Qualitative limitations: Only intervention completers were interviewed (selection bias), non-completers' perspectives missing- Integration limitations: Joint display matrix focused on key outcomes/themes, potential oversight of minor convergent/divergent findings
12	Generalizability	Discuss the generalizability (transferability) of mixed methods findings	- Quantitative generalizability: Limited to CKD patients aged 18-65 years with smartphone proficiency in a single tertiary hospital- Qualitative transferability: Findings on implementation barriers (measurement burden, digital fatigue) may be transferable to similar digital health interventions for chronic diseases- Mixed methods generalizability: The "measurement paradox" and stage-matched intervention framework may inform digital nursing intervention design for CKD management
13	Ethical Considerations	Disclose ethical approval, informed consent, and protection of participants' rights (e.g., anonymity, confidentiality)	- Ethical approval: Institutional Review Board of the Second Affiliated Hospital of Army Medical University (Approval No. 2022-Research-040-01; revised study design approval No. 2022-Research-515-01)- Informed consent: Written informed consent obtained from all participants- Protection measures: Anonymization of interview data (P+number), confidential storage of audio

No.	GRAMMS Core Items	Specific Description	Your Manuscript Response (Fill in Based on Actual Content)
			recordings and transcripts, double translation with fidelity to protect data integrity
14	Conflict of Interest	State any potential conflicts of interest among authors	The authors declare no relevant financial disclosures or other conflicts of interest.
15	Funding Source	Specify the funding body and grant number	Supported by the Chongqing Municipal Science-Technology Bureau and Health Commission jointly funded project (grant number 2022MSXM026)
